# Comprehensive Analyses of SOX7 Provide Novel Insights on Its Tumor Suppressor Role and Its Target Genes with Therapeutic Implications in Multiple Myeloma

**DOI:** 10.3390/ijms27177648

**Published:** 2026-08-26

**Authors:** Arda Ceylan, Xiaozhou Hu, Tevfik Hatipoğlu, Fenangi Chiara Nainkwi, Kadriye Bahriye Payzın, Güner Hayri Özsan, Itır Şirinoğlu Demiriz, Ömer Şeker, Merve Kakçı, Osman Can Öztürk, Bircan Yılmaz, Elif Yıldız, Athanasia Pavlopoulou, Can Küçük

**Affiliations:** 1 İzmir International Biomedicine and Genome Institute, Dokuz Eylül University, İzmir 35330, Türkiye; ardaceylanyl@gmail.com (A.C.); elif.yildiz@ibg.edu.tr (E.Y.); athanasia.pavlopoulou@ibg.edu.tr (A.P.); 2 İzmir Biomedicine and Genome Center, İzmir 35330, Türkiye; 3 Department of Medical Biochemistry, Faculty of Medicine, Dokuz Eylül University, İzmir 35330, Türkiye; xiaozhou.hu@deu.edu.tr; 4 Department of Medical Biology, Faculty of Medicine, Dokuz Eylül University, İzmir 35330, Türkiye; nainkwic50@gmail.com (F.C.N.); bircan.yilmaz@deu.edu.tr (B.Y.); 5 Graduate School of Health Sciences, Dokuz Eylül University, İzmir 35330, Türkiye; 6 Department of Hematology, Faculty of Medicine, İzmir Katip Çelebi University, İzmir 35620, Türkiye; bahriyepayzin@gmail.com; 7 Department of Hematology, Faculty of Medicine, Dokuz Eylül University, İzmir 35330, Türkiye; hayri.ozsan@gmail.com (G.H.Ö.); omer.seker@deu.edu.tr (Ö.Ş.); merveeyahsi@gmail.com (M.K.); osmancanozturkdr123@gmail.com (O.C.Ö.); 8 Department of Internal Medicine, Faculty of Medicine, İstanbul Health and Technology University, İstanbul 34445, Türkiye; dritir@hotmail.com

**Keywords:** SOX7, multiple myeloma, tumor suppressor gene, whole-transcriptome sequencing, ChIP-Seq, Wnt/β-catenin pathway, panobinostat

## Abstract

Multiple myeloma (MM) is an incurable hematological malignancy. *SOX7*, located within the recurrently deleted 8p23.1 region in MM, is suggested to act as a tumor suppressor. We characterized *SOX7* through genetic, epigenetic, and functional analyses in MM cell lines. *SOX7* was frequently silenced due to deletion and/or promoter hypermethylation. Ectopic *SOX7* expression in KMS-18 and MM.1S cell lines caused a progressive decline in *SOX7*-transduced cells and induced G1 cell cycle arrest and/or apoptosis. Although *SOX7* re-expression did not enhance bortezomib efficacy, treatment with the pan-histone deacetylase inhibitor panobinostat induced G1 arrest, promoted apoptosis, and increased *SOX7* expression in MM.1S cells. Whole-transcriptome sequencing identified G1/S progression-related Wnt/β-catenin pathway genes as major SOX7-regulated targets, while ChIP-Seq analysis revealed widespread genomic SOX7 occupancy in MM.1S. Flow cytometric analysis of permeabilized bone marrow tumor cells from newly diagnosed and relapsed MM patients demonstrated generally low SOX7 protein expression. Collectively, these results indicate that *SOX7* functions as a tumor suppressor in MM, and its inactivation promotes cell cycle progression. The anti-myeloma effects of panobinostat in MM.1S cells may be partially mediated through *SOX7* induction.

## 1. Introduction

Multiple myeloma (MM) is a common hematological malignancy characterized by the clonal proliferation of malignant plasma cells within the bone marrow [1]. MM accounts for approximately 1–2% of all cancers and roughly 10–15% of hematologic malignancies, contributing to about 2% of cancer-related deaths worldwide [1,2]. Although therapeutic advances in proteasome inhibitors (e.g., bortezomib), immunomodulatory drugs (IMiDs; e.g., lenalidomide and thalidomide), and pan-histone deacetylase (HDAC) inhibitors have significantly improved patient outcomes, MM remains incurable due to inevitable drug resistance and disease relapse [3]. Therefore, identifying novel molecular mechanisms underlying MM progression is essential for developing more effective therapeutic strategies.

MM pathogenesis is driven by a complex interplay of genetic and epigenetic alterations that dysregulate key signaling pathways controlling plasma cell proliferation and survival [4]. Among these, aberrant activation of the canonical Wnt/β-catenin signaling is a well-established feature of MM and is driven by increased Wnt production, bone marrow microenvironmental interactions, or the silencing of Wnt antagonists [5]. The consequent accumulation of β-catenin promotes the transcription of oncogenic genes involved in tumor cell cycle proliferation and survival [6]. Genome-wide studies have identified numerous tumor suppressor genes that are genetically or epigenetically inactivated in MM, such as *GPX3*, *RBP1*, *SPARC*, *TGFBI*, and *SAMSN1* [7,8].

*SOX7* (SRY-box transcription factor 7), a member of the SOX-F transcription factor family, has recently emerged as a potential tumor suppressor in MM. It is transcriptionally downregulated in both diagnostic and relapsed MM cases through promoter hypermethylation and recurrent deletions in the 8p23.1 chromosomal region [9,10,11]. *SOX7* is frequently downregulated across multiple cancers and functions as a tumor suppressor primarily by antagonizing Wnt/β-catenin signaling. Through a direct interaction with β-catenin, SOX7 disrupts the β-catenin/TCF-mediated transcription of oncogenic targets involved in cell-cycle progression and survival, such as *Cyclin D1*, *c-Myc*, and *COX-2* [12]. Consequently, the restoration of *SOX7* expression in several malignancies inhibited proliferation and induced cell-cycle arrest and apoptosis, consistent with the worse clinical outcomes in cases with lower SOX7 expression [13,14,15,16,17,18]. Although these findings suggest that *SOX7* may play an important role in MM biology, its downstream transcriptional targets and functional mechanisms in MM remain poorly understood. Interestingly, the pan-HDAC inhibitor panobinostat has been reported to restore *SOX7* expression in solid tumors through epigenetic reactivation, ultimately leading to cell death [17]. Nonetheless, it is unclear whether *SOX7* contributes to its therapeutic efficacy in MM.

In the present study, we comprehensively investigated the tumor-suppressive role of SOX7 in MM by characterizing its genetic and epigenetic inactivation and determining the functional effects of its restoration on cell proliferation and apoptosis in cell lines, as well as evaluating its protein expression and its association with patient survival using bone marrow tumor samples. Through whole-transcriptome and chromatin immunoprecipitation sequencing (ChIP-seq) analyses, we identified transcriptional targets of *SOX7*. In addition, we examined the potential relationship between *SOX7* expression and the cellular response to the clinically relevant agents bortezomib and panobinostat to assess the therapeutic potential of *SOX7* in MM.

## 2. Results

### 2.1. SOX7 Is Deleted and Hypermethylated in MM Cell Lines

**Figure 1:**
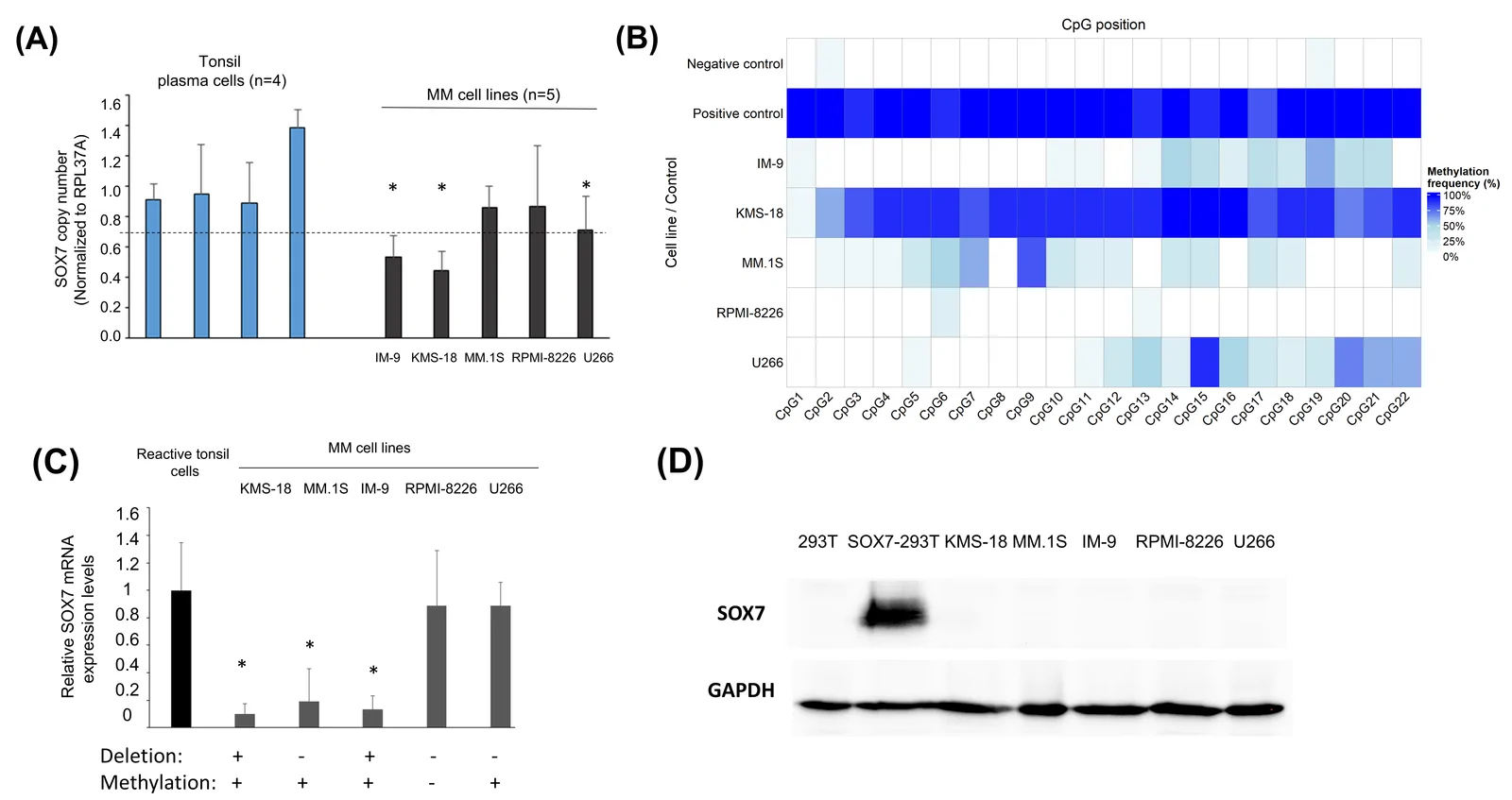
*SOX7* is deleted and hypermethylated in MM cell lines. (**A**) *SOX7* gene copy number in MM cell lines. *SOX7* copy numbers were normalized to *RPL37A*. Plasma cell samples isolated from four different tonsils without copy number abnormalities were used as the control. Data are presented as mean ± s.d. of four independent experiments, each conducted with replicate amplifications (*n* = 8) for both *SOX7* and *RPL37A* apart from two tonsil plasma cell samples that were included in two experiments. *: *p* < 0.05 compared to tonsil plasma cell control samples. (**B**) Epigenetic heatmap showing *SOX7* promoter CpG methylation frequencies determined by bisulfite sequencing in the negative control, positive control, and MM cell lines. The negative control consisted of peripheral blood cells from a healthy donor, whereas the positive control was EpiTect methylated and bisulfite-converted human control DNA. Methylation frequencies are represented using a blue color scale, with white denoting the unmethylated state and progressively darker shades of blue indicating increasing methylation frequency. (**C**) *SOX7* mRNA expression levels in MM cell lines determined by qRT-PCR. The presence or absence of *SOX7* deletion and promoter methylation is indicated by plus (+) and minus (−) signs, respectively. *: *p* < 0.05 versus reactive tonsil cell samples. (**D**) Representative Western blot image from three independent experiments showing SOX7 protein expression levels in MM cell lines. SOX7-HEK293T: positive control consisting of HEK293T cells obtained 48 h after transfection with the SOX7 expression vector. Twenty-five micrograms of whole-cell lysate were loaded per lane. GAPDH was used as loading control.

To identify genetic and epigenetic alterations in MM cell lines, we evaluated the *SOX7* copy number and promoter methylation status. qPCR analysis revealed the monoallelic deletion of *SOX7* in the IM-9 and KMS-18 cell lines, whereas MM.1S, RPMI 8226 and U266 cell lines did not show a significant decrease in the *SOX7* copy number compared to reactive tonsil plasma cells (Figure 1A). Next, *SOX7* promoter methylation across 22 CpG dinucleotides was assessed by bisulfite sequencing. As expected, the negative control sample exhibited either no or minimal methylation (Figure 1B), whereas the positive control sample was fully methylated (Figure 1B). Quantitative analysis revealed extensive *SOX7* promoter methylation in KMS-18 cells; intermediate methylation in IM-9, MM.1S and U266 cells; and minimal methylation in RPMI 8226 cells (Figure 1B).

### 2.2. SOX7 Is Underexpressed in MM Cell Lines at the Transcript and Protein Levels

Next, we investigated whether *SOX7* underexpression in MM cell lines was associated with gene deletion and/or hypermethylation. qRT-PCR analysis showed that *SOX7* mRNA expression was markedly reduced in three MM cell lines compared with the reactive tonsil cells (Figure 1C). Western blot analysis further demonstrated that SOX7 protein expression was undetectable in all MM cell lines examined (Figure 1D).

### 2.3. Ectopic SOX7 Expression Leads to Negative Selection Pressure in MM Cell Lines

**Figure 2:**
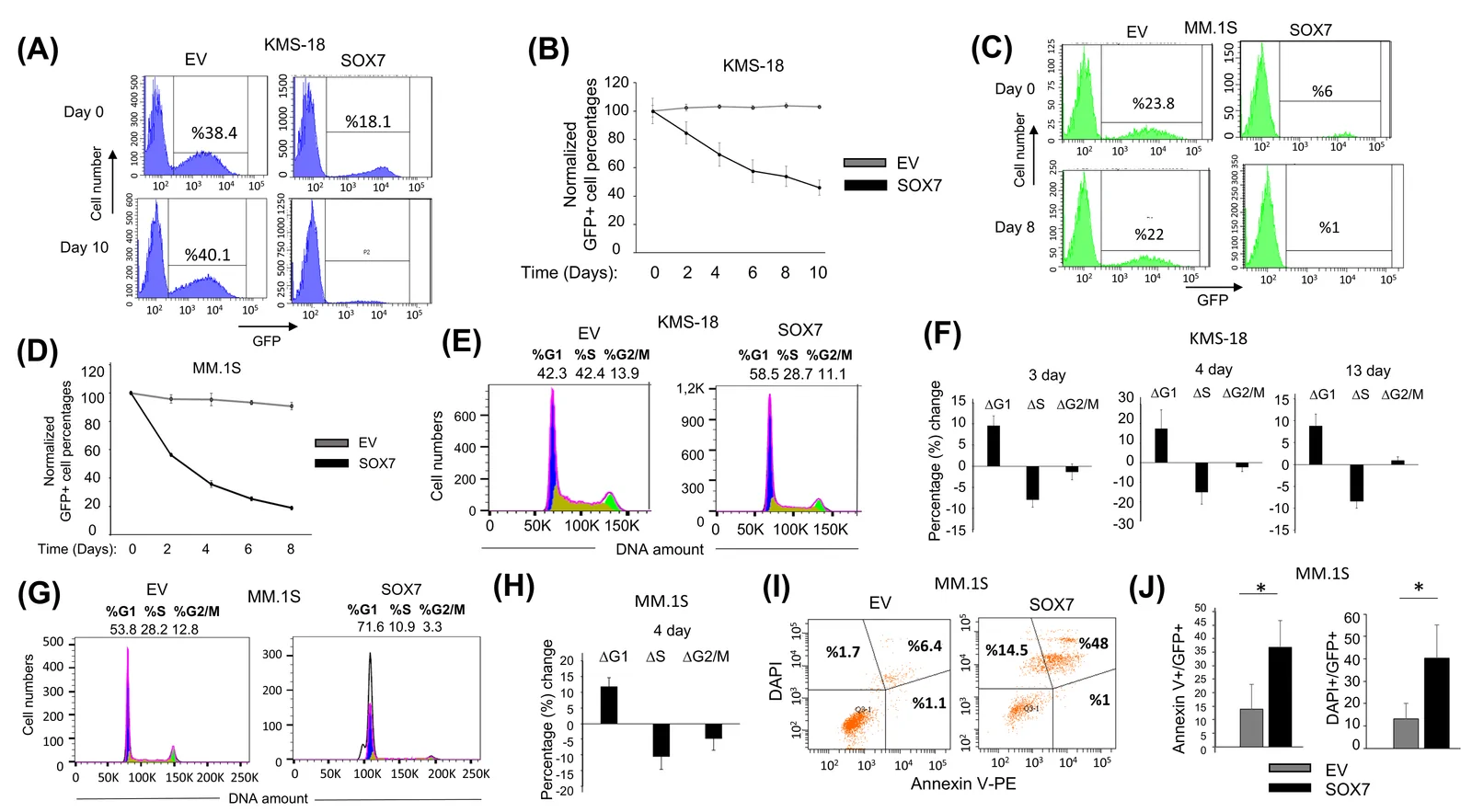
Ectopic *SOX7* leads to negative selection pressure, G1 cell cycle arrest and/or apoptosis in MM cell lines. (**A**) Representative FACS plots showing the percentage of GFP+ cells at the initial analysis and 10 days later in EV- or *SOX7*-transduced KMS-18 cells. (**B**) Normalized percentage of GFP+ cells at consecutive time points in EV- or *SOX7*-transduced KMS-18 cells. (**C**) Representative FACS plots showing the percentages of GFP+ cells at the initial analysis and 8 days later in EV- or *SOX7*-transduced MM.1S cells. (**D**) Normalized percentage of GFP+ cells at consecutive time points in EV- or *SOX7*-transduced MM.1S cells. Normalization was performed by dividing the percentages of GFP+ cells at each subsequent time point by that at Day 0, corresponding to 4 days post-transduction. (**E**) Representative cell cycle plots of EV (left)- or SOX7 (right)-transduced KMS-18 cells four days post-transduction. (**F**) Graphs showing changes in cell cycle distribution in SOX7-transduced KMS-18 cells compared with EV-transduced cells at three, four, and 13 days post-transduction. (**G**) Representative cell cycle plots of EV (left)- or SOX7 (right)-transduced MM.1S cells four days post-transduction. (**H**) Graph showing the percentages of SOX7- and EV-transduced MM.1S cells four days post-transduction in each cell cycle phase. The average values and standard deviations shown for the KMS-18 cell line in panel (F) represent biological replicates, whereas those shown for the MM.1S cell line in panel (H) represent two independent experiment with biological replicates. (**I**) Representative FACS plots of EV- or *SOX7*-transduced MM.1S cells stained with Annexin V-PE and DAPI four days post-transduction. (**J**) Graphs comparing Annexin V+ (left) and DAPI+ (right) cells within the GFP-gated populations of EV- or *SOX7*-transduced MM.1S cells four days post-transduction. EV: PMIG; SOX7: SOX7-PMIG. Values represent the mean ± standard deviation in experimental, biological, and technical replicates (*n* = 8). *: *p* < 0.01.

To address whether *SOX7* functions as a tumor suppressor in MM cell lines, we performed a GFP competition assay by ectopically expressing *SOX7* in two *SOX7* non-expressing MM cell lines and monitoring the proportion of transduced cell populations over regular time intervals. The ectopic expression of *SOX7* in KMS-18 cells resulted in a progressive decline in the percentage of GFP+ cells, whereas the proportion of GFP+ cells in empty vector (EV)-transduced cells remained largely unchanged (Figure 2A,B). Similarly, the proportion of GFP+ MM.1S cells expressing ectopic *SOX7* decreased progressively over time, while the GFP+ cell population in EV-transduced cells were similar to those at the initial time point (Figure 2C,D).

### 2.4. Ectopic SOX7 Expression Causes G1 Phase Cell Cycle Arrest in MM Cell Lines

In order to investigate the mechanisms underlying the tumor suppressor effect observed in the GFP competition assay, we examined whether the re-expression of *SOX7* induces cell cycle arrest. Following the ectopic expression of *SOX7* in the KMS-18 cells, cell cycle analysis (Figure 2E) consistently revealed an increase in the proportion of cells in the G1 phase, with a concomitant decrease in the S phase of the cell cycle at three, four, and 13 (Figure 2F) days post-transduction. Similarly, ectopic expression of *SOX7* in MM.1S cells resulted in an increased and decreased proportion of cells in the G1 and S phase, respectively (Figure 2G,H).

### 2.5. The Influence of Ectopic SOX7 Expression on Apoptosis in KMS-18 and MM.1S Cells

Next, we performed apoptosis analyses in the KMS-18 cell line (Figure S1A,B) to determine whether apoptosis also contributes to the negative selection following ectopic *SOX7* expression. Compared with EV-transduced KMS-18 cells, no significant change in the percentage of annexin V+ cells was observed in *SOX7*-transduced KMS-18 cells at three (Figure S1C) and four (Figure S1D) days post-transduction. We then repeated the apoptosis analyses in the MM.1S cells (Figure 2I). In contrast to KMS-18 cells, ectopic *SOX7* expression significantly increased the percentages of annexin V+ (Figure 2J, left) and DAPI+ (Figure 2J, right) cells compared with EV-transduced control cells four days after transduction.

### 2.6. Ectopic SOX7 Expression Does Not Increase Bortezomib Sensitivity in MM Cell Lines

To determine whether *SOX7* can increase the anti-myeloma effectiveness of bortezomib, we treated two *SOX7*-transduced MM cell lines with two different concentrations of bortezomib for 48 h based on the predetermined EC50 values (Figure S2). Following the transduction of KMS-18 cells with either the empty vector (Figure S3A) or *SOX7* (Figure S3B), apoptosis was assessed after treatment with either bortezomib or only DMSO (Figure S3C,D). Compared with the DMSO-treated control cells (Figure S3E), no increase in apoptosis was observed in *SOX7*-transduced KMS-18 cells treated with 10 nM (Figure S3F) or 12 nM (Figure S3G) bortezomib. Similarly, MM.1S cells were transduced with the empty vector (Figure S4A) or *SOX7* (Figure S4B) and subsequently treated with either DMSO (Figure S4C,D) or bortezomib. As expected, ectopic *SOX7* expression increased apoptosis in DMSO-treated MM.1S cells (Figure S4E). However, compared with the DMSO-treated MM.1S cells, treatment with 4 nM (Figure S4F) or 5 nM (Figure S4G) bortezomib had no additive or synergistic increase in the apoptosis in cells expressing ectopic *SOX7*.

### 2.7. Panobinostat Increases SOX7 mRNA Levels in MM.1S but Not in the KMS-18 Cell Line

To determine whether the anti-myeloma effects of panobinostat involve SOX7, we examined changes in *SOX7* transcript levels in KMS-18 and MM.1S cell lines (Figure S5A). The treatment of KMS-18 cells with 50, 100 or 200 nM panobinostat for 24 h either had no effect (100 nM) or produced a modest increase in the *SOX7* transcript levels compared with untreated or DMSO-treated KMS-18 cells (Figure S5B). In contrast, treatment of MM.1S cells with the same concentrations resulted in marked increase in *SOX7* transcript levels, particularly at 50 nM, compared with untreated or DMSO-treated cells (Figure S5C).

### 2.8. Panobinostat Induces S Phase Arrest and Apoptosis in KMS-18 Cells

**Figure 3:**
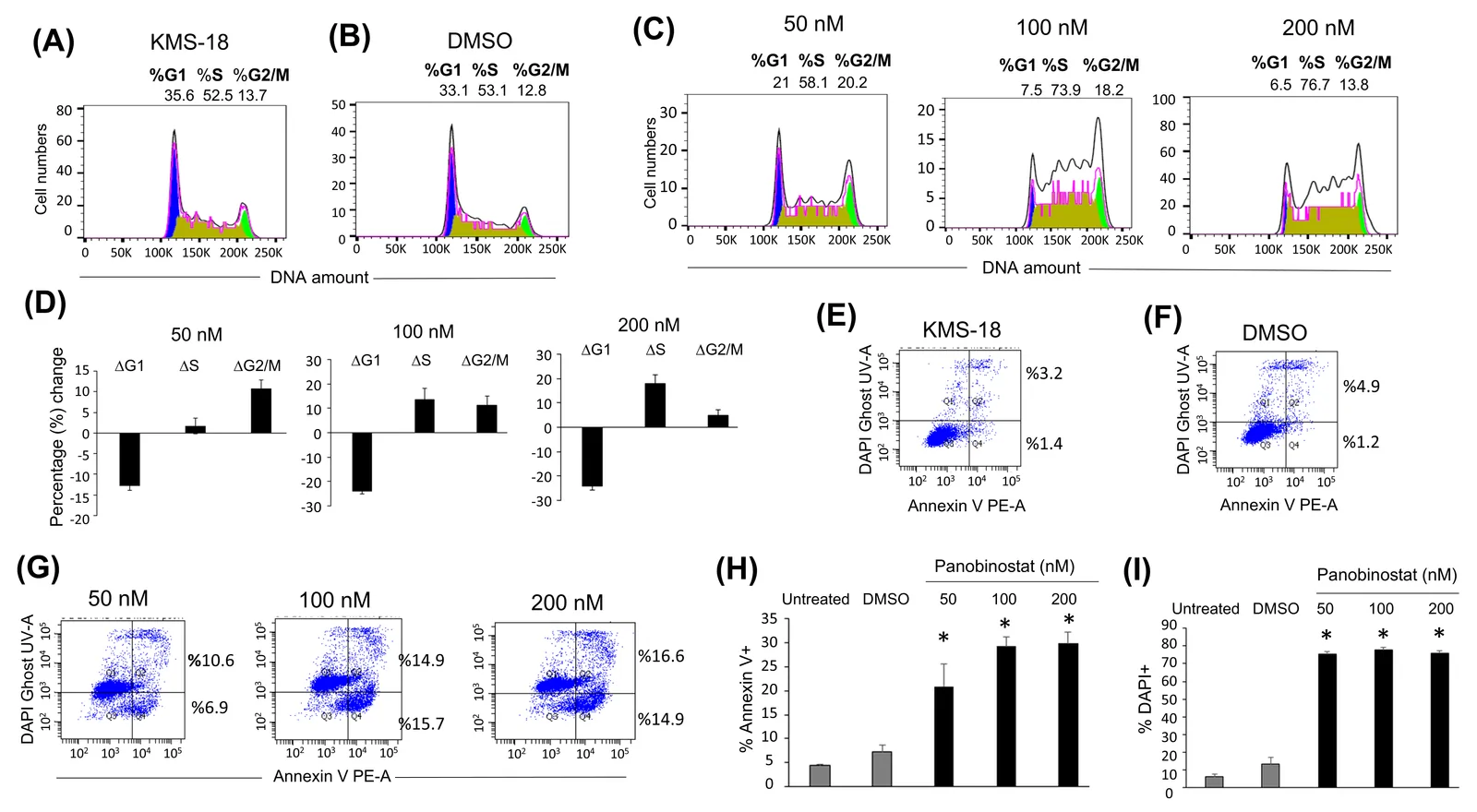
The effect of panobinostat on cell cycle and apoptosis in KMS-18 cells. Representative FACS plots showing the percentages of cells in the G1, S and G2/M phases in untreated (**A**), DMSO-treated (**B**), or panobinostat-treated (**C**) KMS-18 cells. (**D**) Bar graphs showing changes in cell cycle distribution following treatment with 50 nM, 100 nM, or 200 nM panobinostat compared with only DMSO-treated cells. Data are presented as the mean ± SD of biological replicates. Representative FACS graphs of Annexin V and DAPI staining in untreated (**E**), DMSO-treated (**F**), or panobinostat-treated (**G**) KMS-18 cells for 24 h. Bar graphs showing Annexin V-positive (**H**) and DAPI-positive (**I**) cells in control (gray) and panobinostat-treated (black) cells. *: *p* < 0.01 versus untreated and DMSO-treated controls.

Cell cycle analysis of panobinostat-treated KMS-18 cells (Figure 3A–C) revealed a notable increase in the proportion of cells in the S phase with a concomitant decrease in the G1 phase, compared with the DMSO-treated cells (Figure 3D). Next, we assessed the effect of panobinostat on cell survival through Annexin V and DAPI staining (Figure 3E–G). Panobinostat treatment significantly increased the percentages of both Annexin V+ (Figure 3H) and DAPI+ (Figure 3I) cells at all concentrations tested compared to untreated or DMSO-treated cells.

### 2.9. Panobinostat Induces G1 Cell Cycle Arrest and Apoptosis in MM.1S Cells

Next, MM.1S cells were treated with 50 nM, 100 nM or 200 nM panobinostat to evaluate its effect on cell cycle progression (Figure S6A–C). Compared with the DMSO-treated control, panobinostat treatment increased the proportion of cells in the G1 phase, with a concomitant decrease in the S and G2/M phases at all tested concentrations (Figure S6D). We next examined the effect of panobinostat on cell survival (Figure S7A–C). Treatment with panobinostat significantly increased the percentage of Annexin V+ MM.1S cells at all tested concentrations compared both with the untreated and DMSO-treated controls (Figure S7D). In addition, DAPI+ cells were markedly increased following treatment with 100 nM or 200 nM panobinostat compared with the control groups (Figure S7E).

### 2.10. SOX7 Regulates Common and Distinct mRNAs and lncRNAs in KMS-18 and MM.1S Cells

**Figure 4:**
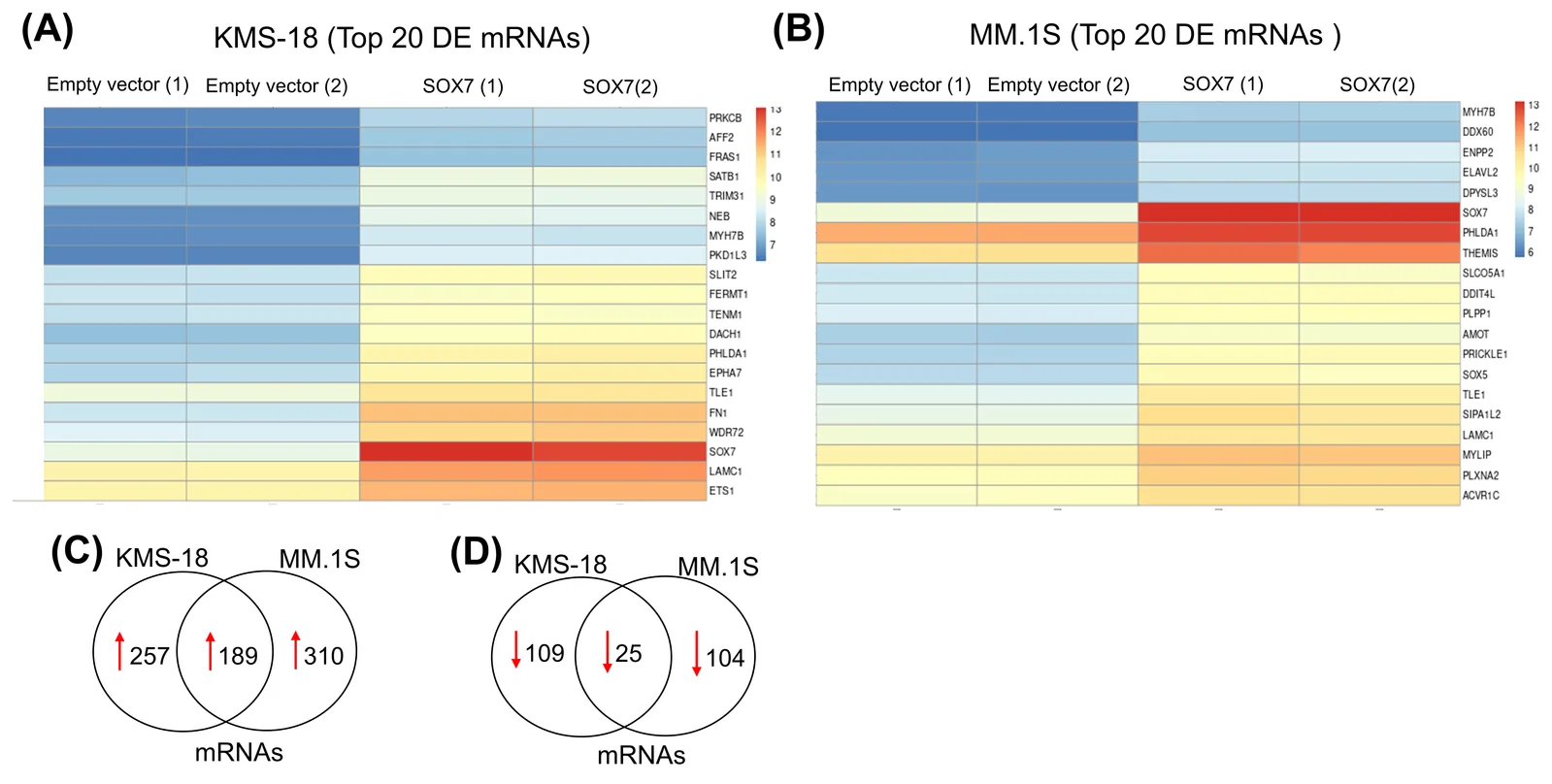
Protein-coding genes transcriptionally regulated by *SOX7* in MM cell lines. Heatmaps showing the top 20 differentially expressed protein-coding genes in KMS-18 (**A**) and MM.1S (**B**) cells. The color scale indicates fold change. Venn diagrams generated using Venny 2.1. show the numbers of significantly upregulated (**C**) and downregulated (**D**) mRNAs identified in KMS-18 or MM.1S cells based on WTS. Upward or downward arrows indicate upregulation or downregulation of transcripts, respectively, following ectopic *SOX7* expression.

**Figure 5:**
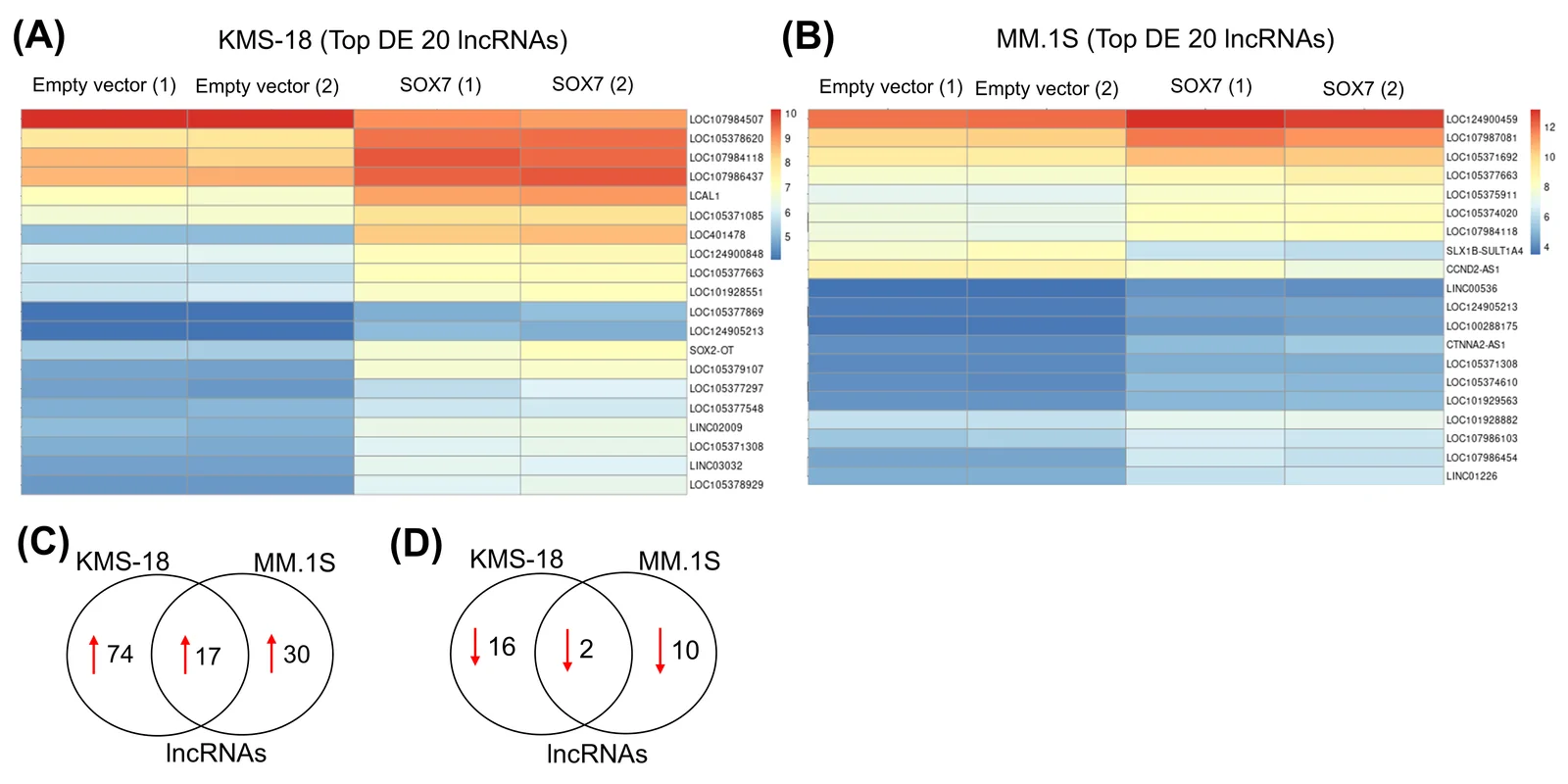
LncRNAs transcriptionally regulated by *SOX7* in MM cell lines. Heatmaps showing the top 20 differentially expressed lncRNAs in the KMS-18 (**A**) and MM.1S (**B**) cells. The color scale indicates fold change between *SOX7*-transduced and EV-transduced cells. Venn diagrams showing the number of significantly upregulated (**C**) or downregulated (**D**) lncRNAs identified in MM.1S or KMS-18 based on WTS. Upward and downward arrows indicate upregulated and downregulated transcripts, respectively, following ectopic *SOX7* expression.

Because the *SOX7*-mediated tumor suppressor functions are likely mediated through its activity as a transcription factor, we investigated *SOX7*-regulated transcripts by performing whole-transcriptome sequencing (WTS) following ectopic *SOX7* expression. As a proof-of-principle, *SOX7* was among the most highly upregulated genes in both *SOX7*-transduced KMS-18 (Figure 4A) and MM.1S (Figure 4B) cells compared to the EV-transduced controls. Of note, all of the top 20 differentially expressed protein-coding genes were upregulated in both MM cell lines following ectopic *SOX7* expression (Figure 4A,B). Consistent with this observation, the number of significantly upregulated mRNAs (Figure 4C) greatly exceeded the number of downregulated transcripts in both cell lines (Figure 4D). Overall, we identified 189 commonly upregulated (Figure 4C) and 25 commonly downregulated mRNAs (Figure 4D) in MM.1S and KMS-18 cells. In contrast to the mRNA profile, several of the top differentially expressed lncRNAs were downregulated following *SOX7* overexpression in both cell lines (Figure 5A,B). Overall, seventeen of these lncRNAs were significantly upregulated by *SOX7* in both KMS-18 and MM.1S cells (Figure 5C), whereas only two lncRNAs were commonly downregulated (Figure 5D). The differentially expressed mRNAs and lncRNAs are listed in Tables S1 and S2.

### 2.11. SOX7-Regulated Signaling Pathways Are Associated with Cell Cycle Regulation in KMS-18 and MM.1S Cells

**Figure 6:**
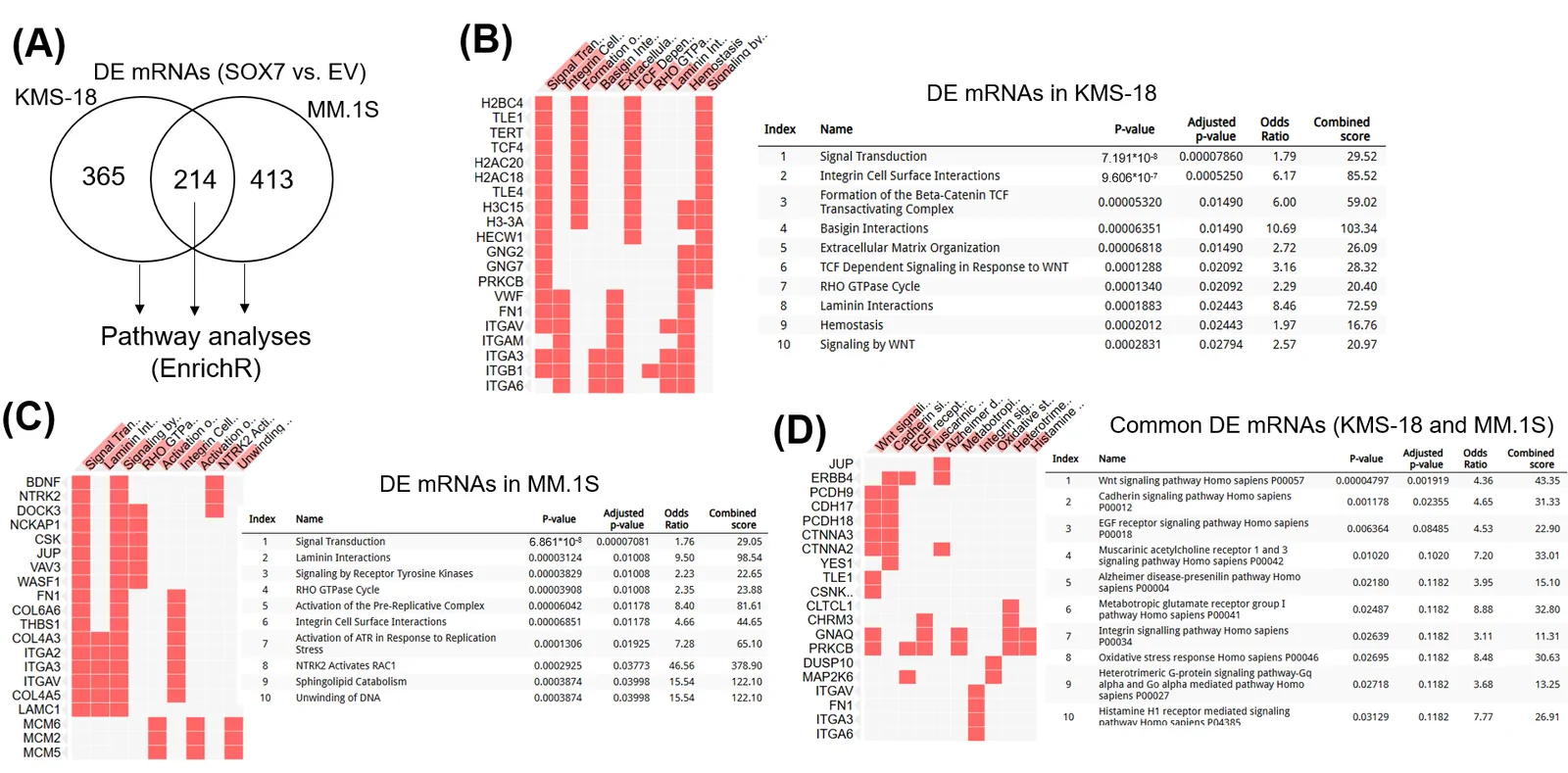
Signaling pathways associated with ectopic *SOX7* expression in MM cells. (**A**) Venn diagram showing the number of DE mRNAs in KMS-18 and/or MM.1S cells (padj ≤ 0.05). Clustergrams (left) and corresponding tables (right) show the top 10 enriched signaling pathways based on differentially expressed protein-coding genes in KMS-18 (**B**), MM.1S (**C**), and genes commonly DE in both KMS-18 and MM.1S cells (**D**). Pathways are ranked according to increasing adjusted *p* value. *SOX7* was excluded from the input gene list because it is ectopically expressed. Pathway analysis in panel B was performed on PANTHER 2016 database, whereas analyses in panels (**C**,**D**) were based on Reactome Pathways 2024. The order of the signaling pathways is the same in the clustergrams from left to right and the corresponding tables from top to bottom. DE: differentially expressed.

To determine whether *SOX7*-regulated genes are associated potentially with oncogenic signaling pathways, we performed pathway enrichment analysis using significantly differentially expressed protein-coding genes in KMS-18 and/or MM.1S cell lines. Differentially expressed protein-coding genes, excluding *SOX7*, were provided as inputs for enrichment analysis with the Enrichr platform [19] (Figure 6A). Among the top 10 significantly enriched signaling pathways in KMS-18 cells, signal transduction, integrin–cell surface interactions, RHO GTPase cycle, and WNT signaling pathways were the most prominent (Figure 6B). Similarly, signal transduction, RHO GTPase cycle, and integrin–cell surface interactions were among the top ten over-represented pathways in MM.1S cells (Figure 6C). In addition, pathways associated with DNA replication initiation and replication stress were significantly enriched in MM.1S cells. Importantly, analysis of genes commonly differentially expressed in both KMS-18 and MM.1S cells identified WNT and cadherin signaling among the most significantly enriched pathways (Figure 6D). Although the EGF receptor signaling pathway did not reach statistical significance (padj = 0.08), it ranked as the third highest over-represented pathway in the analysis (Figure 6D). In contrast, no signaling pathways were significantly enriched among the differentially expressed lncRNAs identified in KMS-18 and/or MM.1S cells.

### 2.12. Cross-Validation of Transcriptional Changes in CCND2 and DUSP6 with qRT-PCR

**Figure 7:**
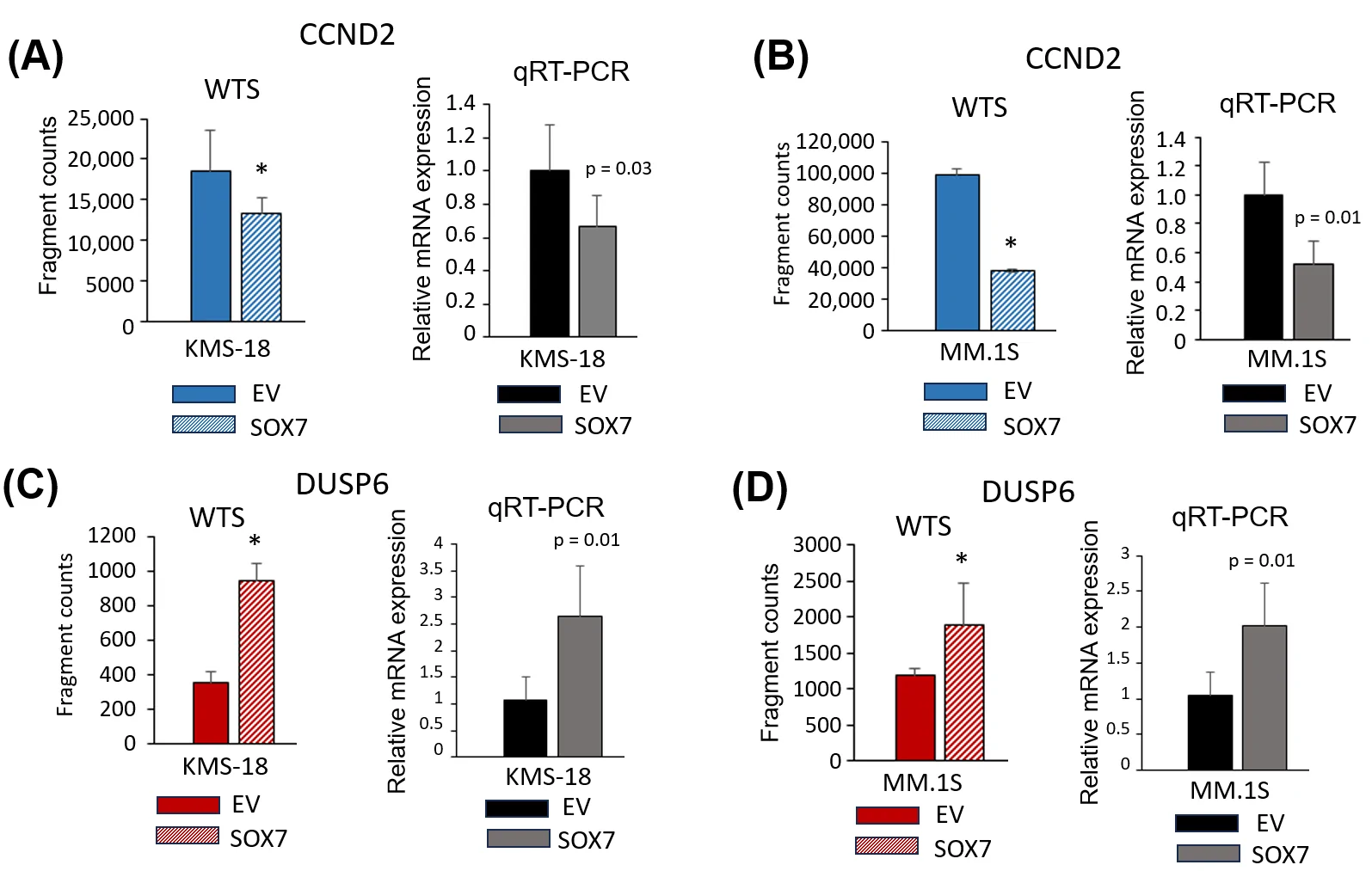
qRT-PCR validation of SOX7-mediated *CCND2* and *DUSP6* expression changes detected by WTS. Bar graphs showing transcriptional downregulation of *CCND2* in EV- or *SOX7*-transduced, GFP-sorted KMS-18 (**A**) and MM.1S (**B**) cell lines determined by WTS (left) or qRT-PCR (right). Bar graphs showing transcriptional upregulation of *DUSP6* in EV- or *SOX7*-transduced, GFP-sorted KMS-18 (**C**) and MM.1S (**D**) cell lines two days post-transduction. qPCR analyses of *DUSP6* and *CCND2* were performed using two and three independent experiments, respectively, each with duplicates. *RPL37A* was used as the housekeeping gene. Data are presented as mean ± SD. qRT-PCR *p* values were computed using *t*-test. *: *P*adj < 0.05 obtained using DESeq2. EV: Empty vector; WTS: Whole-transcriptome sequencing.

As an independent validation of the SOX7-related transcriptional changes observed in the Wnt/β-catenin pathway, we conducted qRT-PCR for *CCND2* and *DUSP6* using empty vector or *SOX7*-transduced KMS-18 and MM.1S cell lines. Consistent with the WTS results, SOX7 transcriptionally downregulated *CCND2* in KMS-18 (Figure 7A) and MM.1S (Figure 7B) cell lines. Similarly, the SOX7-mediated *DUSP6* upregulation detected by WTS in both MM cell lines was cross-validated via qRT-PCR in the KMS-18 (Figure 7C) and MM.1S (Figure 7D) cell line.

### 2.13. Ectopic SOX7 Binds to Promoters and Distal Genomic Regions in KMS-18 and MM.1S Cells

**Figure 8:**
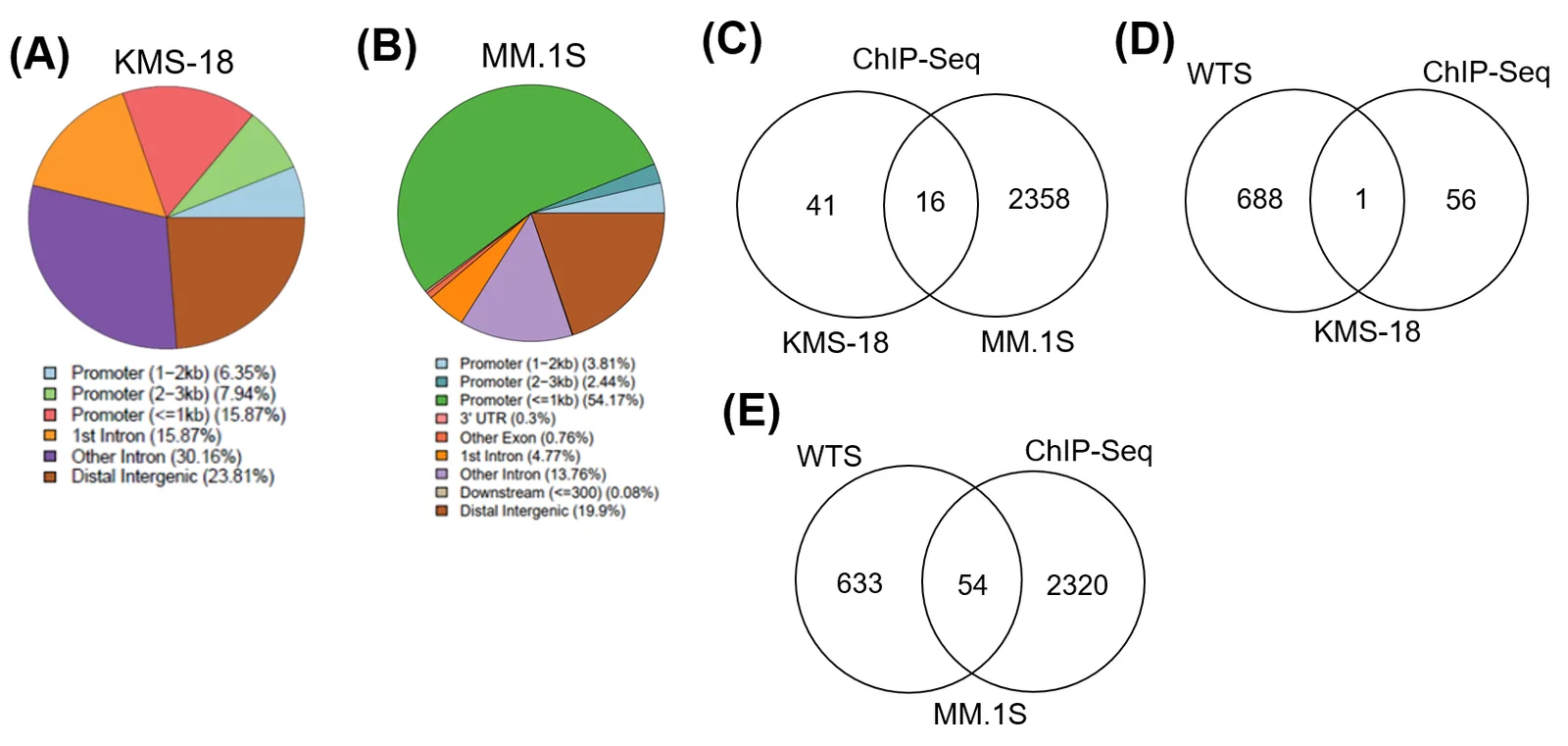
SOX7 occupancy across genomic regions and its association with DE genes in KMS-18 and MM.1S cell lines. Distribution of SOX7 ChIP-Seq peaks across genomic regions in KMS-18 (**A**) and MM.1S (**B**) cells. (**C**) Venn diagram depicting the overlap of genes annotated to SOX7-binding sites between KMS-18 and/or MM.1S cells, determined using ChIPseeker. Venn diagrams showing the overlap between genes associated with SOX7-binding sites and DE genes identified by WTS in KMS-18 (**D**) and MM.1S (**E**) cells. DE: Differentially expressed.

Next, we identified genomic regions directly occupied by *SOX7* in KMS-18 and MM.1S cells following ectopic SOX7 expression. In KMS-18 cells, SOX7 binding was detected across various genomic regions, including but not limited to promoter regions (Figure 8A). Interestingly, ectopically expressed *SOX7* occupied a substantially higher proportion of promoter regions in MM.1S cells than in KMS-18 cells (Figure 8B). By annotating the identified ChIP-Seq peaks, we found that only 16 genes adjacent to SOX7 binding sites were common in both KMS-18 and MM.1S cells (Figure 8C). Unexpectedly, only one differentially expressed gene in KMS-18 cells harbored a nearby SOX7-binding site (Figure 8D). On the other hand, 54 of the 687 DE genes identified in MM.1S cells were associated with a neighboring SOX7-binding site (Figure 8E).

### 2.14. *SOX7* Protein Expression and Its Clinical Association with Patient Survival in MM

To evaluate SOX7 protein expression levels in MM, we measured SOX7 positivity by flow cytometry in bone marrow samples collected from diagnostic and relapsed MM patients (Figure S8A,B). In general, the proportion of SOX7+ cells was low among the CD38+/CD138+/CD19- MM plasma cell population (Figure S8C). Moreover, SOX7 protein expression was lower in relapsed samples compared to diagnostic samples (Figure S8C). Next, we investigated the relationship between SOX7 protein expression and clinical outcome in patients with MM. No significant differences in overall (Figure S9A) or progression-free (Figure S9B) survival were observed between MM patients with low or high SOX7 protein expression.

## 3. Discussion

Recurrently deleted genomic regions are frequently enriched for tumor suppressor genes [20,21]. Although the deletion of the 8p23.1 locus has long been documented in newly diagnosed MM cases, the characterization of candidate tumor suppressor genes within this region remains largely unresolved to the best of our knowledge. Recent evidence has highlighted the potential of *SOX7* to act as a tumor suppressor gene that is silenced through the classical “two-hit” mechanisms, involving concurrent genetic deletion and promoter hypermethylation, in MM and related plasma cell neoplasms [11], as well as in hematologic malignancies such as acute myeloid leukemia (AML) [22]. Consistent with these findings, we demonstrate that *SOX7* inactivation results from a combination of copy number losses and promoter hypermethylation, leading to marked transcriptional and protein downregulation. Of note, the absence of detectable SOX7 protein expression across all examined MM cell lines, including those without complete gene deletion or extensive promoter methylation, suggests that additional regulatory mechanisms (e.g., post-transcriptional regulation, histone modifications, enhanced protein degradation, etc.) may also contribute to *SOX7* silencing.

The restoration of *SOX7* function via ectopic delivery imposed a negative selection pressure on both KMS-18 and MM.1S cell models, suggesting that its presence prevents sustained MM cell growth. This suppression is mainly mediated by G1 cell cycle arrest rather than direct cytotoxicity. Similar anti-proliferative activities, whereby *SOX7* suppresses tumor growth by halting cell cycle progression, have been reported in colorectal [18], lung [16], and hepatocellular cancers [23].

Interestingly, *SOX7* restoration elicited cell line-specific differential responses. Whereas *SOX7* induced both G1 arrest and apoptosis in MM.1S cells, KMS-18 cells primarily underwent cell cycle arrest with minimal apoptosis. These differences are likely attributed to the molecular heterogeneity of MM rather than differences in *SOX7* itself [24], as distinct genetic and epigenetic landscapes across MM cell lines may influence the array of *SOX7*-responsive genes and interacting cofactors. Consistent with this notion, transcriptome analyses identified numerous cell line-specific, *SOX7*-regulated genes. Notably, several pro-apoptotic genes were selectively induced in MM.1S cells, partially explaining the apoptosis observed exclusively in this cell line. These included *BCL2L11 (BIM)*, which encodes a BCL-2 family member that antagonizes anti-apoptotic proteins in order to trigger mitochondrial outer membrane permeabilization and caspase activation [25], as well as *DAPK1* and its homolog *DAPK2*, calcium/calmodulin-regulated serine/threonine kinases that act downstream of IFN-γ, TNF-α, and Fas signaling to mediate both caspase-dependent apoptosis and autophagy-linked cell death [26].

Whole-transcriptome sequencing and functional enrichment analyses revealed that *SOX7* exerts its tumor-suppressive footprint in MM by restraining cell proliferation largely through the modulation of key components of the canonical Wnt/β-catenin pathway. Specifically, *SOX7* upregulates the Wnt antagonists *TLE1*, which encodes a transcriptional corepressor that displaces β-catenin from TCF/LEF complexes [27], and *PRICKLE1*, a suppressor of Dishevelled-mediated signaling [28]. Conversely, *SOX7* downregulates Wnt-associated genes, including *CCND2*, a downstream target of β-catenin/TCF4 signaling [29] and key regulator of G1/S progression [30], as well as *CXXC4* (*IDAX*), which encodes a Dishevelled-binding protein previously reported to antagonize Wnt/β-catenin signaling [31]. Likewise, the expression of the oncogenic lncRNA *SNHG3*, which promotes cancer progression through the activation of Wnt/β-catenin signaling [32], is suppressed in both cell lines. Because *DUSP6* is a direct transcriptional target gene of Wnt/β-catenin signaling [33], it acts as a negative feedback regulator of the RAS-ERK pathway, thereby attenuating cancer cell signaling [34]. Therefore, the upregulation of *DUSP6* suggests that SOX7 alters Wnt-mediated transcription in a way that suppresses oncogenic RAS-ERK signaling and, ultimately, inhibits myeloma cell growth.

ChIP-Seq identified the genome-wide binding profile of SOX7 in MM cells. Interestingly, SOX7 occupied a vastly higher number of promoter regions in MM.1S compared to KMS-18 cells, thus resulting in a much larger overlap between SOX7 binding sites and differentially expressed genes in MM.1S. In contrast, only a limited number of direct SOX7 target genes were identified in KMS-18 cells, despite extensive transcriptional changes. These observations suggest that SOX7 regulates gene expression through both direct promoter binding and indirect transcriptional programs, with the dominance of each mechanism depending on the cellular context. Such context-dependent regulatory behavior is a well-recognized feature of SOX family members and may be due to differences in chromatin accessibility and/or interactions with specific cofactors [35,36].

In this study, the relationship between the anti-cancer agent panobinostat and *SOX7* was investigated. Panobinostat is a non-selective pan-HDAC inhibitor that promotes histone acetylation, resulting is a more permissive chromatin structure that facilitates RNA polymerase access to DNA and transcriptional activation of tumor suppressor and other anti-cancer genes [37]. As expected, panobinostat increased *SOX7* mRNA levels in a context-dependent manner, with a more pronounced induction in MM.1S than in KMS-18 cells, likely due to the combined effect of monoallelic deletion and heavy baseline promoter hypermethylation at the *SOX7* locus in KMS-18 cells.

Ectopic *SOX7* expression induced observable apoptosis in MM.1S cells but not in KMS-18 cells. However, panobinostat increased apoptosis in both cell lines, indicating that its antitumor activity is only partially mediated by *SOX7.* This is likely due to panobinostat’s capacity to target diverse epigenetic networks; through its broad HDAC inhibitory activity, it can simultaneously activate multiple pro-apoptotic genes and suppress diverse oncogenic pathways alongside the *SOX7* axis.

In contrast, *SOX7* restoration did not enhance sensitivity to the proteasome inhibitor bortezomib in either MM cell line, suggesting that the two exert their anti-myeloma effects through distinct mechanisms. While SOX7 mainly interferes with cell cycle progression by modulating transcriptional networks, bortezomib induces proteotoxic stress, endoplasmic reticulum stress, the activation of the unfolded protein response [38], and ultimately apoptosis. Taken together, these findings indicate that panobinostat and *SOX7* share overlapping transcriptional tumor-suppressive programs in MM, whereas bortezomib induces cell death through the disruption of protein homeostasis.

The association between SOX7 and the clinical outcomes in MM patients was also investigated, and generally low SOX7 protein expression was observed in both newly diagnosed and relapsed patients, with lower expression in relapsed cases. The fact that SOX7 expression was not associated significantly with overall or progression-free survival in our cohort is probably attributed to the limited sample size which may have reduced statistical power. Therefore, larger independent patient cohorts are required to determine the prognostic or predictive value of SOX7 in MM.

Several limitations of this study should be acknowledged. Although functional experiments were conducted in two MM cell lines, in vivo validation was beyond the scope of this work. Moreover, the numerous candidate SOX7 target genes identified by ChIP-seq require further experimental verification. Future studies should also investigate the functional role of SOX7 in other hematological malignancies, such as acute or chronic myeloid leukemia, to further clarify its impact on hematologic cancers.

In summary, we provide compelling evidence that *SOX7* functions as a *bona fide* tumor suppressor in multiple myeloma and is frequently silenced by dual deletion and promoter hypermethylation. The functional restoration of *SOX7* suppresses cell growth by driving G1 cell cycle arrest and/or context-dependent apoptosis through the orchestrated transcriptional regulation of multiple genes—particularly those implicated in the Wnt/β-catenin pathway or cell cycle regulatory networks. Additionally, our findings suggest that *SOX7* induction may contribute to the anti-myeloma activity of panobinostat in a context-dependent manner. However, future studies including SOX7 knock-down in panobinostat-treated MM cell lines are needed to clarify the role of SOX7 upregulation on the anti-myeloma effect observed. Taken together, these results demonstrate that *SOX7* expression is tightly regulated by diverse mechanisms, underscoring its biological importance in maintaining plasma cell homeostasis and highlighting downstream *SOX7* targets as promising candidates for future clinical investigations.

## 4. Materials and Methods

### 4.1. Cell Lines Used in This Study

Five well-characterized multiple myeloma (MM) cell lines (IM-9, KMS-18, MM.1S, RPMI 8226, and U266), as well as the HEK293T cell line, were included in this study. KMS-18, IM-9, MM.1S, and HEK293T cell lines were kindly provided by Dr. Anna Scuto (City of Hope Medical Center, Duarte, CA, USA). RPMI 8226 and U266 cell lines were kindly provided by Dr. Asuman Sunguroğlu (Ankara University, Ankara, Türkiye). HEK293T cells were cultured in DMEM medium supplemented with 10% fetal bovine serum (FBS), 1% Penicillin/Streptomycin and 1% L-Glutamine. MM cell lines were cultured in RPMI culture medium supplemented with 10% FBS, 1% Penicillin/Streptomycin and 1% L-Glutamine. All cell lines were cultured in an incubator at 37 °C containing 5% CO_2_. The absence of mycoplasma contamination in the cell lines used in this study was confirmed using the Mycoplasma PCR Detection Kit (Applied Biological Materials Inc., Richmond, BC, Canada) according to the manufacturer’s instructions.

### 4.2. Tonsil Plasma Cell DNA Samples as Non-Cancer Controls

Plasma cells were obtained from the tonsil tissue samples of four obstructive sleep apnea (OSA) patients, as described previously [39] through FACS by gating on CD38^+^, IgD^−^, and CD77^−^ cells. Genomic DNA samples were isolated using the AllPrep DNA/RNA Mini Kit (Qiagen, Hilden, Germany) per the manufacturer’s instructions.

### 4.3. Copy Number Analyses of SOX7 in MM Cell Lines

Genomic DNA (gDNA) was isolated from MM cell lines using the QIAamp DNA Mini Kit (Qiagen, Hilden, Germany) per the manufacturer’s instructions. *SOX7* copy number analyses were performed by quantitative PCR using either Roche LightCycler 480 (Roche Diagnostics, Basel, Switzerland) or an ABI 7500 Fast Real-Time PCR System with QuantiTect SYBR Green qPCR Master Mix (Qiagen, Hilden, Germany). *SOX7* copy numbers were normalized to those of *RPL37A*. The optimal annealing temperatures for the *SOX7* and *RPL37A* genomic qPCR primer pairs were determined with gradient PCR. CD38^+^/IgD^−^/CD77- plasma cells obtained from four different obstructive sleep apnea cases were used as the normal *SOX7* copy number reference. The nucleotide sequences of the qPCR primers are as follows:

SOX7-genomic-qPCR-F: 5′-GTTAGGACACACCCGAACAA-3′, SOX7-genomic-qPCR-R: 5′-CCTCGTCGAAAGGCAAAGA-3′, RPL37A-genomic-qPCR-F: 5′-GCTTTCACATCCCTGACAATAAG-3′, and RPL37A-genomic-qPCR-R: 5′-CTCGTCTCTTCATCTTGGTCTATAA-3′.

### 4.4. Bisulfite Sequencing of the SOX7 Promoter in MM Cell Lines

The nucleotide sequence spanning the *SOX7* transcription start site (TSS ± 1 kb) was retrieved from the NCBI nucleotide database (https://blast.ncbi.nlm.nih.gov/Blast.cgi, accessed on 15 March 2022). The visualisation of this 2 kb region showed that the majority overlapped with a CpG island (Figure S10). Two primer pairs for bisulfite sequencing were designed with the MethPrimer program (http://www.urogene.org/methprimer/, accessed on 15 March 2022). The names and nucleotide sequences of these primer pairs are as follows: SOX7-bisulfite-seq-1 primer pair: 5′-GTTGTTGGGAGTTTATTTTTGGTT-3′ (forward) and 5′-CTCACTCACCCAACATCTTACTAAAC-3′ (reverse); SOX7-bisulfite-seq-2 primer pair: 5′-TTAATTAGGTGGTTGAGAATTAGAA-3′ (forward) and 5′-ATTAACCATAAACCCCTCAAAACA-3′ (reverse). The negative control sample with an unmethylated *SOX7* promoter was generated by collecting peripheral blood from a healthy donor. Briefly, 8 mL of peripheral blood was mixed with 25 mL BG1 buffer solution, followed by gDNA isolation using the Paxgene Blood DNA Kit (Qiagen, Hilden, Germany) per the manufacturer’s instructions. Bisulfite modification of the negative control DNA and gDNA isolated from five MM cell lines was performed using the EZ DNA Methylation-Direct™ Kit (Zymo Research, Irvine, CA, USA), according to the manufacturer’s protocol. Given that CpG dinucleotides were reported previously to be absent around the *SOX7* TSS in normal tissues [40], DNA from the healthy donor was used as the unmethylated negative control. Fully methylated and bisulfite-converted human control DNA from the EpiTect PCR Control DNA Set (Qiagen, Hilden, Germany) served as the positive control. *SOX7* promoter CpG methylation frequencies across MM cell lines were quantitatively visualized using an epigenetic heatmap generated in R (v4.2.2) with the ComplexHeatmap (v2.22.0) package.

### 4.5. qRT-PCR

*Quantification of SOX7 transcript levels*: Total RNA was isolated from MM cell lines using the RNeasy Mini Kit (Qiagen, Hilden, Germany) per the manufacturer’s instructions. Total RNA concentration and purity were determined with the NanoDrop spectrophotometer, and RNA integrity was evaluated by electrophoresis on a 1% TAE agarose gel. Real-time PCR was performed using QuantiTect SYBR Green qPCR Master Mix (Qiagen Inc., Hilden, Germany) on an 7500 Fast Real-Time PCR System (Applied Biosystems, Foster City, CA, USA). Reactive tonsil cells obtained from a non-cancer individual were used as the normalization control. *SOX7* mRNA expression levels were normalized to those of the *RPL37A* reference gene [41]. Relative transcript expression was calculated using the comparative Ct (ΔΔCt) method [42]. The optimum annealing temperatures of the *SOX7* and *RPL37A* qRT-PCR primer pairs were determined by gradient PCR. The primer sequences were as follows: SOX7-qRT-PCR-F: 5′-GCCGAGCTCAGCAAGAT-3′; SOX7-qRT-PCR-R: 5′-CGGCCGGTACTTGTAGTTG-3′; RPL37A-qRT-PCR-F: 5′-AGTCGGGATCGTCGGTAAATA-3′; RPL37A-qRT-PCR-R: 5′-GCCACAGAAAGAGCAAGTGTA-3′.

*Quantification of CCND2 and DUSP6 mRNA levels in SOX7-transduced KMS-18 and MM.1S cell lines*: Previously published primer pairs amplifying *CCND2* [43] and *DUSP6* [44] cDNA were synthesized by Macrogene. The optimum primer annealing temperatures were determined using gradient PCR on MM.1S cDNA samples. The total RNA samples of EV- or *SOX7*-transduced, GFP-sorted KMS-18 and MM.1S cells used for WTS experiments were also utilized for qRT-PCR analysis. Total RNA was reverse-transcribed to cDNA with the QuantiTect Reverse Transcription Kit (Qiagen, Hilden, Germany) according to the manufacturer’s instructions. Real-time qPCR experiments were conducted on a BIORAD CFX™ Duet RT-PCR system or a LightCycler^®^ 480 Instrument II for 40 cycles using QuantiTect SYBR Green qPCR Master Mix. The following primer pairs were used for these qRT-PCR experiments alongside with the same *RPL37A* primer pair introduced above: CCND2-qRT-PCR-F: 5′- CCAACACAGACGTGGATTGT-3′; CCND2-qRT-PCR-R: 5′-GTTCATCCTCCGACTTGGA-3′; DUSP6-qRT-PCR-F: 5′-AGCTCAATCT- GTCGATGAACG-3′; DUSP6-qRT-PCR-R: 5′-GCGTCCTCTCGAAGTCCAG-3′.

### 4.6. Western Blot

Because none of the MM cell lines expressed endogenous SOX7 protein, HEK293T cells were transiently transfected with the SOX7-PMIG plasmid using TurboFect (Thermo Fisher Scientific, Waltham, MA, USA) per the manufacturer’s protocol to generate positive control samples. Cells cultured in 10% FBS/DMEM were lysed 24 h post-transfection in RIPA buffer (Thermo Fisher Scientific, Waltham, MA, USA) supplemented with protease/phosphatase inhibitors (Thermo Fisher Scientific, Waltham, MA, USA). Cell lysates were centrifuged at 14,000× *g* for 15 min at 4 °C, and protein concentrations were determined by NanoDrop A280; protein lysates were stored at −80 °C. Equal amounts of protein were mixed with Laemmli buffer (Bio-Rad, Hercules, CA, USA) containing β-mercaptoethanol, denatured at 95 °C for 5 min, and separated on 12% resolving/4% stacking SDS-PAGE gels. A PageRuler protein ladder (Thermo Fisher Scientific, Waltham, MA, USA) served as the molecular weight marker; electrophoresis was performed at 80 mA for 1 h. Proteins were transferred onto methanol-activated PVDF membranes (Bio-Rad, Hercules, CA, USA) using a wet transfer system at 80V for 90 min under ice cooling conditions. To prevent non-specific binding, membranes were blocked overnight at 4 °C in Tris-buffered saline with Tween-20 (TBST) containing 5% non-fat milk. The blots were then incubated for 1 h at room temperature (RT) with gentle agitation in 1% milk/TBST containing anti-SOX7 (Cat. no. AF2766; R&D Systems, Minneapolis, MN, USA) or anti-GAPDH (Cat. no. AF5718; R&D Systems, Minneapolis, MN, USA) primary antibodies. Following three 10 min washes in TBST, the membranes were incubated with a horseradish peroxidase (HRP)-conjugated rabbit anti-goat secondary antibody (Thermo Fisher Scientific, Waltham, MA, USA) diluted 1:1000 in blocking buffer for 1 h at RT with agitation. After three additional 10 min TBST washes, immunoblots were visualized using Clarity Western ECL substrate (Bio-Rad, Hercules, CA, USA) after a 5 min incubation in the dark. Chemiluminescence signals and colorimetric molecular weight ladders were captured using a ChemiDoc MP imaging system (Bio-Rad, Hercules, CA, USA).

### 4.7. Generation of the SOX7 Retroviral Expression Construct

The pcDNA3.1-SOX7 plasmid containing the human *SOX7* coding sequence was obtained as dried DNA on filter paper and dissolved in elution buffer. As the amount of the pcDNA3.1-SOX7 plasmid DNA was insufficient, it was heat-shock transformed into competent bacteria, and then plated onto ampicillin-containing LB agar Petri dishes. A single bacterial colony was incubated in LB medium for plasmid amplification followed by collection of bacteria through centrifugation. High-purity pcDNA3.1-SOX7 plasmid DNA was extracted at a sufficient concentration using the QIAprep Spin Miniprep Kit (Qiagen, Hilden, Germany). The SOX7-PMIG-NotI-F and SOX7-PMIG-SalI-R primer pair was optimized by gradient PCR using the pcDNA3.1-SOX7 plasmid as the template DNA. After that, the *SOX7* coding sequence was amplified with high fidelity from the pcDNA3.1-SOX7 plasmid using PfuUltra II Fusion HS DNA Polymerase (Agilent Technologies, Santa Clara, CA, USA). The amplified product containing NotI and SalI restriction sites was purified from agarose gel using the QIAquick Gel Extraction Kit (Qiagen, Hilden, Germany). The purified SOX7 amplicon was double digested with the NotI-HF (New England Biolabs, Ipswich, MA, USA) and SalI-HF (New England Biolabs (Ipswich, MA, USA) restriction enzymes and purified using the QIAquick PCR Purification Kit (Qiagen, Hilden, Germany). The PMIG retroviral vector was digested sequentially with NotI-HF and SalI-HF, followed by purification after each digestion using the QIAquick PCR Purification Kit. The digested SOX7 insert and the PMIG plasmid vector were ligated with T4 DNA ligase (New England Bioloabs, Ipswich, MA, USA; Cat. no.: M0202S). Following heat shock transformation, competent bacteria were plated on LB agar containing ampicillin and incubated. Positive colonies were identified by colony PCR. Plasmid DNA from positive clones from bacteria cultured in 30 mL ampicillin-containing LB medium was purified using the QIAprep Spin Miniprep Kit. Insertion of the *SOX7* coding sequence was further verified through restriction mapping with SalI and NotI enzymes. Finally, Sanger sequencing was performed by GATC Biotech-Eurofins to confirm lack of any mutation in the cloned sequence of SOX7-PMIG plasmid. The sequencing primers were as follows: SOX7-PMIG-Sanger-1-F: 5′-GCCAAGGACGAGAGGAAAC-3′; SOX7-PMIG-Sanger-1-R: 5′-GTACGTGTCCACACTGCTC-3′; SOX7-PMIG-Sanger-2-F: 5′-CCCTGCCGGAGAAGAGA-3′; SOX7-PMIG-Sanger-2-R: 5′-TGACACACTGTAGCTGTTGTAG-3′. The obtained sequences were analyzed using NCBI BLASTn (2.17.0) [45] and Unipro UGENE v41.0 [46].

### 4.8. Reconstitution of SOX7 Expression in MM Cell Lines

HEK293T cells were seeded into 10 cm round dishes, twenty-four hours before transfection. HEK293T cells were co-transfected with the empty PMIG vector or the SOX7-PMIG construct together with the PCL-Ampho [47] plasmid one day after seeding. Twenty-four hours post-transfection, the culture medium was replaced with 3 mL fresh medium. Polybrene (Sigma Aldrich, St Louis, MO, USA) was added at a final concentration of 10 μg/mL to the suspended cells. In brief, 5 × 10^5^ cells were placed into 12-well plates and subjected to spinoculation at 1500 rpm for 90 min at 4 °C. After that, cells were incubated at 37 °C with 5% CO_2_. Cells were centrifuged at 300× *g* for 5 min seven hours after transduction. The pellets were then resuspended in 4 mL of regular cell culture medium and transferred to T25 cell culture plates for incubation. Forty-eight hours after transduction with either the empty vector or SOX7-PMIG, the percentage of GFP+ cells was determined by FACS. Flow cytometry data were analyzed with BD FACS Diva 8.0 (BD Biosciences, San Jose, CA, USA). The ectopic expression of SOX7 protein was further verified by Western blot (Figure S11). The overall experimental workflow for the GFP competition, cell cycle, and apoptosis assays performed in MM cells with ectopically expressed *SOX7* expression is illustrated in Figure S12.

### 4.9. GFP Competition Assay

Forty-eight hours after the retroviral transduction of KMS-18 and MM.1S cells with either PMIG or SOX7-PMIG, the percentage of GFP+ cells was determined using the BD LSRFortessa™ Cell Analyzer (BD Biosciences, San Jose, CA, USA). Flow cytometry data were analyzed with the BD FACS Diva 8.0 software.

### 4.10. Cell Cycle Analyses

Cell cycle distribution was analyzed with DAPI (BioLegend, San Diego, CA, USA, Cat no: 422801) staining 4 days post-transduction. Briefly, cells were fixed with the True-Nuclear Transcription Factor Fix (BioLegend, San Diego, CA, USA) for 1 h at RT in the dark. Cells were then centrifuged at 400× *g* for 5 min at RT and resuspended in 1 mL of 1x PBS for 15 min to allow rehydration. Following the additional centrifugation and removal of the supernatant, cells were stained with 3 μM DAPI prepared in 200 μL permeabilization buffer and incubated at RT for 15 min. Cell cycle analysis was performed by flow cytometry, and the distribution of GFP+ cells among different cell cycle phases was determined using the FlowJo v10.7.1 software (BD Biosciences, San Jose, CA, USA).

### 4.11. Apoptosis Assay

Apoptosis rates of the PMIG- or SOX7-transduced MM cells were determined with the PE Annexin V Apoptosis Detection Kit I (BD Biosciences, San Diego, CA, USA, Cat. no.: 559763) according to the manufacturer’s recommendations, except that 100 μL instead of 400 μL 1x Binding Buffer was used. DAPI (BioLegend, San Diego, CA, USA) was added to a final concentration of 3.75 μM. Flow cytometry data were analyzed with the BD FACS Diva 8.0 software.

### 4.12. Evaluation of the Effect of Ectopic SOX7 Expression on Bortezomib Sensitivity in MM Cell Lines

Bortezomib powder was dissolved in DMSO (Santa Cruz Biotechnology, Dallas, TX, USA, Cat. no.: sc-358801) to prepare a 1 mM stock solution. Subsequently, this stock was diluted in complete RPMI medium supplemented with 10% FBS, 1% penicillin/streptomycin, and 1% L-glutamine to obtain a 200 nM bortezomib working solution. Bortezomib treatment was initiated 47 h and 54.5 h post-transduction in KMS-18 and MM.1S cells, respectively. Cell numbers were determined using the trypan blue exclusion assay, after which 10,000 cells in 250 μL were seeded into each well of 96-well F-bottom cell culture plates (Greiner Bio-One, Frickenhausen, Germany; Cat. No. 655180) (containing bortezomib (Bio-Techne, Minneapolis, MN, USA, Cat. no.: 7282/5) at varying concentrations (0.5, 1, 1.5, 2, 2.5, or 5 nM). Control groups consisted of cells cultured in 10% FBS RPMI medium containing either DMSO or medium alone. Three biological replicates were included for both the bortezomib-treated and control groups. After incubation at 37 °C for 48 h in a sterile incubator with 5% CO_2_, cell counts were determined using the trypan blue exclusion assay. Cell viability percentages following bortezomib treatment were determined by calculating the percentage of cell death. This was done by dividing the difference in mean viable cell counts between each bortezomib-treated sample and the DMSO control group by the mean cell count of the control group. Following 48 h of bortezomib exposure, changes in the percentages of Annexin V+/GFP+ (early apoptotic) and DAPI+/GFP+ (late apoptotic/dead) cells were quantified.

### 4.13. Investigation of the Effects of Panobinostat on SOX7 Expression, Cell Cycle, and Apoptosis

Powdered panobinostat (1 mg; Bio-Techne, Minneapolis, MN, USA, Cat. no.: 7629/10) was dissolved in 2.86 mL DMSO (Santa Cruz Biotechnology, Dallas, TX, USA, Cat. no.: sc-358801) to prepare a 1 mM stock solution. The resulting solution was sterile-filtered using a syringe equipped with a 0.2 μm filter. Subsequently, panobinostat was diluted to 200 nM concentration in complete cell culture medium consisting of RPMI supplemented with 10% FBS, 1% penicillin/streptomycin, and 1% L-glutamine. This 200 nM solution was further serially diluted with complete culture medium to obtain additional treatment concentrations of 100 nM and 50 nM. For the vehicle control group, DMSO was added to the complete culture medium at a concentration identical to that used in the 200 nM panobinostat preparation. KMS-18 or MM.1S cells were seeded at a density of 1.2 × 10^4^ cells/mL in 4.5 mL volumes per well. Experimental groups were treated with 50 nM, 100 nM, or 200 nM panobinostat, while control groups received either culture medium alone or DMSO-supplemented medium. Following a 24 h incubation period, qRT-PCR, cell cycle arrest, and apoptosis experiments were performed.

For *SOX7* transcript quantification, total RNA was isolated using the AllPrep DNA/RNA Micro Kit (Qiagen, Hilden, Germany; Cat. no: 80284) with a double elution to maximize yield. RNA concentration and purity were verified using Thermo Scientific™ NanoDrop 2000. Reverse transcription was performed on 0.5 μg RNA using the QuantiTect Reverse Transcription Kit (Qiagen, Hilden, Germany, Cat. no: 205311). The resulting cDNA was diluted 1:10 and subjected to qPCR using the QuantiTect SYBR Green Master Mix (Qiagen, Hilden, Germany; Cat. no: 204143) on a 7500 Fast Real Time PCR System (Applied Biosystems, Foster City, CA, USA). The same *SOX7* and *RPL37A* primer pairs described in Section 4.5 qRT-PCR subsection were also used here. Melting curve analysis and ΔΔCt calculations were performed using the 7500 Software v2.3, assigning Ct = 40 as threshold to samples showing no amplification.

Cell cycle analysis was conducted using the True-Nuclear Transcription Factor Buffer Set (BioLegend, San Diego, CA, USA; Cat. no: 424401). Cells were initially fixed in 200 μL of 1X Fix Solution for 1 h in dark at RT. Fixed cells were pelleted by centrifugation at 400× *g* for 5 min, and rehydrated in PBS for 15 min. The cells were then stained with 3 μM DAPI (BioLegend, San Diego, CA, USA; Cat. no: 422801) in 1X Perm Buffer prior to FlowJo v10.1 analysis.

Apoptosis was assessed using the BD Pharmingen™ PE Annexin V Apoptosis Detection Kit I (BD Biosciences, San Diego, CA, USA; Cat. no: 559763). Cells were washed with cold phosphate-buffered saline (PBS) at 300× *g* for 5 min at 4 °C. The cell pellets were resuspended in 100 μL of 1× binding buffer and stained with 4 μL of PE Annexin V for 15 min at RT in the dark. Subsequently, 100 μL of 1× binding buffer and 2.5 μL of DAPI (300 μM) were added to the suspension. Samples were analyzed on a BD FACSDiva™ flow cytometer (version 8.0.1). Five conditions were evaluated: medium-only control, DMSO control, and three concentrations of panobinostat, each with biological replicates.

### 4.14. Identification of SOX7 Binding Sites Through ChIP-Seq

KMS-18 and MM.1S cells were transduced with empty PMIG or SOX7-PMIG expression plasmids as previously described. Forty-eight hours post-transduction, GFP+ cells were sorted through a BD FACS Aria III cell sorter. Chromatin immunoprecipitation (ChIP) was performed using 1.0 × 10^5^ GFP+ cells per sample following the Low Cell ChIP Kit protocol (Active Motif, Carlsbad, CA, USA; Cat. no.: 53086). Post-fixation with 37% formaldehyde, chromatin was fragmented via sonication using a Bioruptor Pico (Diagenode, Liège, Belgium; Cat. no.: B01060010) with 20 cycles of 30 s pulses followed by 30 s pauses (total 20 min). Cellular debris was pelleted by centrifugation at 18,000× *g* for 2 min at 4 °C. Next, 180 μL of chromatin-containing supernatant was transferred to fresh tubes and stored at −80 °C. On day 2, Protein G agarose beads were prepared for chromatin pre-clearing. Immunoprecipitation (IP) was then performed using 20 μL anti-SOX7 antibody (R&D Systems, Minneapolis, MN, USA; Cat. no.: AF2766). Day 3 consisted of washes, IP reaction elutions, and cross-link reversal. On day 4, the precipitated DNA was purified and dissolved in low-EDTA TE buffer for storage at −20 °C. DNA library preparations and next-generation sequencing (NGS) experiments were performed step-by-step as follows: (1) End repair of immunoprecipitated DNA fragments; (2) A-tailing (adenine nucleotide addition); (3) Ligation of Illumina adapters; (4) Size selection of the adapter-ligated fragments; (5) PCR amplification. NGS was performed by the Novogene Genome Sequencing Company (Hong Kong, China) using the NovaSeq X Plus Series system using Illumina SBS technology, to generate 150 bp paired-end (PE150) reads. The primary NGS quality control statistics of these ChIP-Seq experiments are available in Table S3.

Genomic regions occupied by SOX7 were identified by the following computational pipeline. Initial raw FASTQ data filtering was conducted by Novogene across three quality control stages: (1) Removal of adapter-contaminated reads; (2) Exclusion of reads with >10% undetermined bases; (3) Filtering of low-quality reads where >50% bases had Q-scores ≤ 5. Subsequent bioinformatic analyses were conducted on the Galaxy public platform (https://usegalaxy.org/, accessed on 15 July 2024): (1) Alignment of raw sequencing reads to the human reference genome hg38 via Bowtie2 [48]; (2) Removal of poorly aligned, low-quality, and PCR-duplicate reads from the resulting BAM files using the BAM filter; (3) Peak calling was executed using MACS2 [49] to identify significant SOX7 binding regions; (4) Genomic annotation of the identified binding sites using ChIPSeeker [50].

### 4.15. Whole-Transcriptome Sequencing

PMIG- or SOX7-PMIG-transduced KMS-18 and MM.1S cells were sorted for GFP+ expression 2 days post-transduction using the BD FACSAria III cell sorter (Figure S13A,B). Total RNA was isolated with the AllPrep DNA-RNA Micro Kit (Qiagen Inc., Hilden, Germany). We evaluated the quality of the total RNA samples with qRT-PCR (Figure S13C,D). Further QC was performed by Novogene through visualisation of 28S and 18S rRNAs in the TAE gel (Figure S13E) as well as analysis using an Agilent 2100 Bioanalyzer (Agilent Technologies, Santa Clara, CA, USA). Whole-transcriptome sequencing (WTS) was performed on biological replicates, comprising a total of 8 samples. The WTS library preparation steps were conducted as follows: first, ribosomal RNA was removed from the total RNA and then precipitated with ethanol. The remaining RNA biomolecules were fragmented, and the first strand cDNA was synthesized using random hexamer primers. dUTPs were replaced with dTTPs in the reaction buffer. After end-repair, A-tailing, adapter ligation, size selection, USER enzyme digestion, PCR amplification, and purification; paired-end 150 bp sequencing was performed to produce ~12 GB of raw data per sample. The basic NGS statistics for WTS are shown in Table S4.

Bioinformatics analyses of differentially expressed transcripts were performed within the RStudio platform (https://posit.co/, (accessed on 1 September 2023)). The quality of the raw NGS data were checked using the FastQC program (v0.11.9). High-quality reads were aligned to the GRCh38 reference human genome using HISAT2 (v2.2.1) [51]. Based on the NCBI RefSeq reference genome annotation, the number of reads matching with exons was quantified using featureCounts (v2.0.3) [52]. For both cell lines, count data from the PMIG- and SOX7-PMIG-transduced groups were compared in order to identify differentially expressed genes was obtained using DESeq2 (v.1.42.0) [53].

### 4.16. Multiple Myeloma Patient Samples and Clinical Information

A total of 64 multiple myeloma (MM) patients diagnosed at the hematology departments of Dokuz Eylül University, İzmir Katip Çelebi University, İstanbul Aydın University, Özel Beykent University, and Şişli Kolan International Hospital were enrolled in this study; bone marrow samples were obtained at the time of diagnosis or relapse. Demographic, clinical, and pathological data, alongside bone marrow samples from these 64 definitively diagnosed MM patients were incorporated. The cohort consisted of 31 males (48.4%) and 33 females (51.6%), with a mean age of 62.2 ± 13.3 years. Patient clinical profiles comprised two primary variable categories: serum electrophoresis values—including immunoglobulin concentrations (IgG, IgA, IgM in mg/dL) and free light chain concentrations (kappa and lambda)—and CRAB criteria parameters, encompassing hemoglobin (g/dL), creatinine (mg/dL), GFR (ml/min), calcium (mg/dL), albumin (g/dL), and LDH levels (U/L). The mean ± standard deviation values for the 64 patients were as follows: IgG 1749.7 ± 1892.0 mg/dL; IgA 431.8 ± 900.0 mg/dL; IgM 39.8 ± 34.0 mg/dL; kappa 440.7 ± 997.7 mg/L; lambda 397.8 ± 922.3 mg/L; kappa/lambda ratio 42.3 ± 94.9; hemoglobin 10.0 ± 2.2 g/dL; creatinine 2.0 ± 2.3 mg/dL; GFR 55.9 ± 35.9 mL/min; calcium 9.8 ± 1.3 mg/dL; albumin 5.3 ± 7.2 g/dL; LDH 235.7 ± 240.0 U/L.

### 4.17. Isolation and Storage of Mononuclear Cells from MM Bone Marrow

MM bone marrow (BM) aspirates were diluted 3-fold with 1x PBS. The diluted BM samples were added into tubes containing Ficoll Paque Plus (Cytiva, Marlborough, MA, USA; Cat. No. GE17-1440-02) in a 3:4 bone marrow/Ficoll to PBS ratio, followed by centrifugation at 400× *g* for 30 min at RT. The mononuclear cell layer was collected and transferred into 15 mL tubes. To wash the cells, an equal or greater volume of 1x PBS was added, and the mixture was centrifuged at 400× *g* for 10 min at RT. The supernatant was discarded, and the cell pellet was resuspended in 5 mL 1x PBS by gentle pipetting. This washing step was repeated following the same procedures. The isolated bone marrow mononuclear cells (BM-MNC) were cryopreserved under sterile conditions in a cell culture cabinet. The cells were resuspended in a freezing medium consisting of 70% FBS, 20% RPMI, and 10% DMSO by gently pipetting up and down 10 times. After that, the cell suspension was transferred into cryovials and kept for 3 min on ice, and placed at −80 °C for 24 h to 7 days. Finally, the cryovials were transferred to a liquid nitrogen tank for long storage until FACS analysis.

### 4.18. Determination of SOX7 Expression in MM Bone Marrow Tumor Cells by FACS

The cryopreserved BM-MNC samples were thawed and stained with CD138-FITC (BioLegend, San Diego, CA, USA; Cat. no: 352304), CD38-BV421 (BioLegend, ,San Diego, CA, USA; Cat. No. 356618), and CD19-APC (BioLegend, San Diego, CA, USA; Cat. No. 302212) according to the manufacturer’s instructions. Cells were then fixed with True-NuclearTM Transcription Factor Buffer Set (BioLegend, San Diego, CA, USA; Cat. No. 424401) for permeabilization. After that, cells were stained with SOX7-PE (BD Biosciences Pharmingen, San Diego, CA, USA; Cat. No. 564695) according to the manufacturer’s protocol, followed by FACS analyses.

### 4.19. Evaluation of the Relationship Between SOX7 Expression and MM Survival

The relationship between SOX7 protein levels in bone marrow tumor cells from newly diagnosed MM patients and overall survival (OS) or progression-free survival (PFS) was examined. The patients were stratified into high- and low-SOX7 expression groups using the median SOX7 protein level as the cutoff. Survival analyses were performed using IBM SPSS Statistics 24 software. For OS analyses, survival time for deceased patients was defined as the interval between the date of diagnosis and the date of death. For living patients or those with unknown survival status, survival time was calculated from the date of diagnosis to the date of the last follow-up. For PFS analyses, the time for the patient group with disease progression was calculated from the date of diagnosis to the either the first documented progression or the last follow-up for patients without documented progression.

### 4.20. Statistical Analysis

Statistical analyses were performed using the *t*-test in Microsoft Office Excel (Microsoft, Redmond, WA, USA), unless otherwise stated. A *p* value <0.05 was considered statistically significant.

## Data Availability

The raw data files of the ChIP-Seq and WTS experiments will be available in the NCBI SRA repository through the following link: https://dataview.ncbi.nlm.nih.gov/object/PRJNA1302653?reviewer=r81nqko5osmkt5rkr2fju2rv9m (accessed on 7 August 2025).

## Supplementary Materials

The following supporting information can be downloaded at https://www.mdpi.com/article/10.3390/ijms27177648/s1.
