# Supplementary material for: Comprehensive Analyses of SOX7 Provide Novel Insights on Its Tumor Suppressor Role and Its Target Genes with Therapeutic Implications in Multiple Myeloma
Source: Int J Mol Sci. 2026 Aug 26;27(17):7648. doi: 10.3390/ijms27177648 (PMC13566877; doi:10.3390/ijms27177648)
Supplement: Supplementary file 1 [file ijms-27-07648-s001.zip › Supplementary figures.pdf]

Figure S1

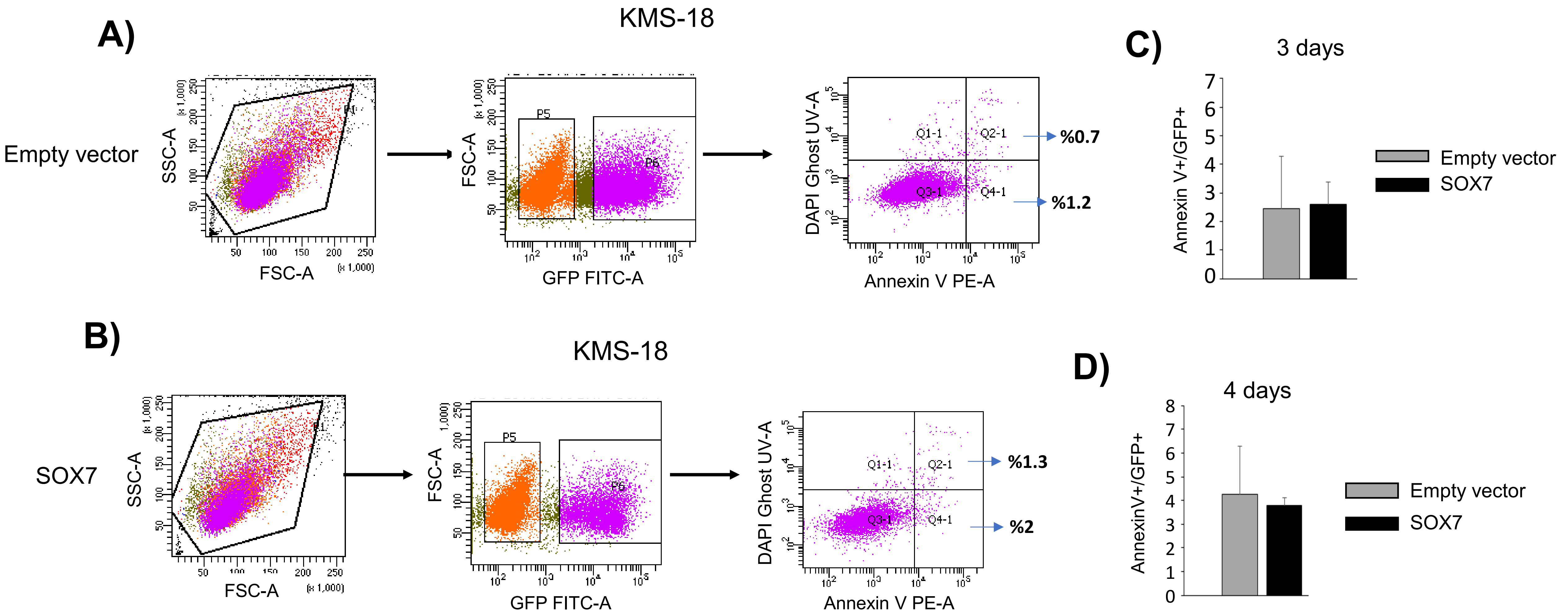

Ectopic SOX7 expression does not lead to apoptosis of KMS-18. Representative FACS plots showing gatings of empty vector (**A**) or SOX7 (**B**) transduced KMS-18 cells 4 days post-transduction to evaluate apoptosis. Comparison of apoptotic cells in empty vector or SOX7 transduced KMS-18 cells determined based on annexin V+/GFP+ cell positivity three (**C**) or four (**D**) days after transduction. Average and standard deviation values represent a combination of two independent experiments with biological replicates. **Empty vector**: PMIG, **SOX7**: SOX7-PMIG.

Figure S2

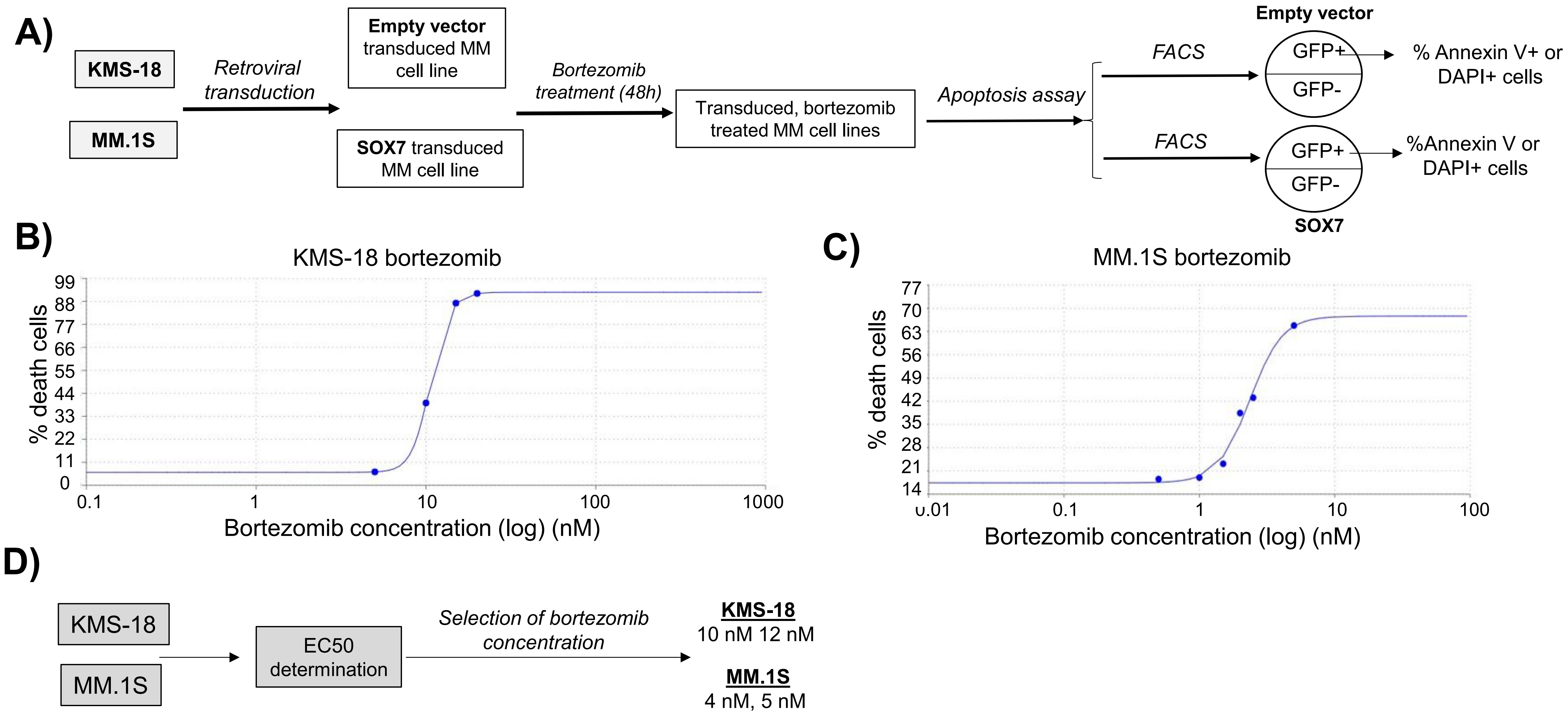

Bortezomib EC50 analyses and graphics in KMS-18 and MM.1S cell lines. A) The workflow followed during investigation of effect of ectopic SOX7 expression on bortezomib sensitivity. The graphs constructed during determination of bortezomib EC50 values for KMS-18 (**B**) and MM.1S (**C**) cell lines. **D)** Workflow showing the strategy for determination of bortezomib treatment doses based on EC50 values.

Figure S3

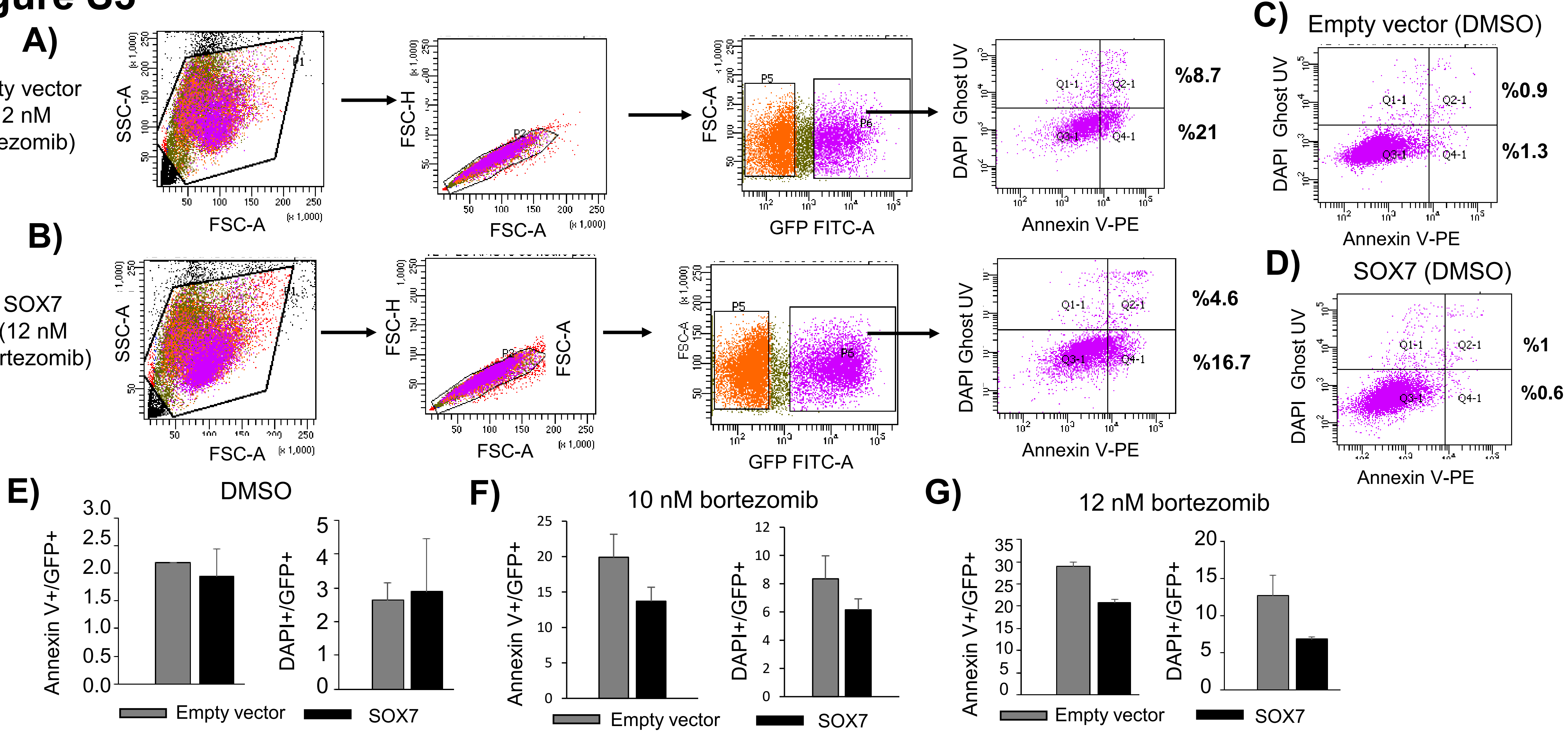

The effect of ectopic SOX7 on bortezomib sensitivity in KMS-18 cells. Representative FACS gatings of empty vector or SOX7 transduced KMS-18 cells treated with 12 nM bortezomib (**A**,**B**) or only DMSO (**C**,**D**) for 48h. Bar graphs comparing Annexin V+ (**left**) or DAPI+ (**right**) cell percentages in EV or SOX7 transduced MM.1S cells in only DMSO (**E**), 10 nM bortezomib (**F**) or 12 nM bortezomib (**G**) treated KMS-18 cells.

Figure S4

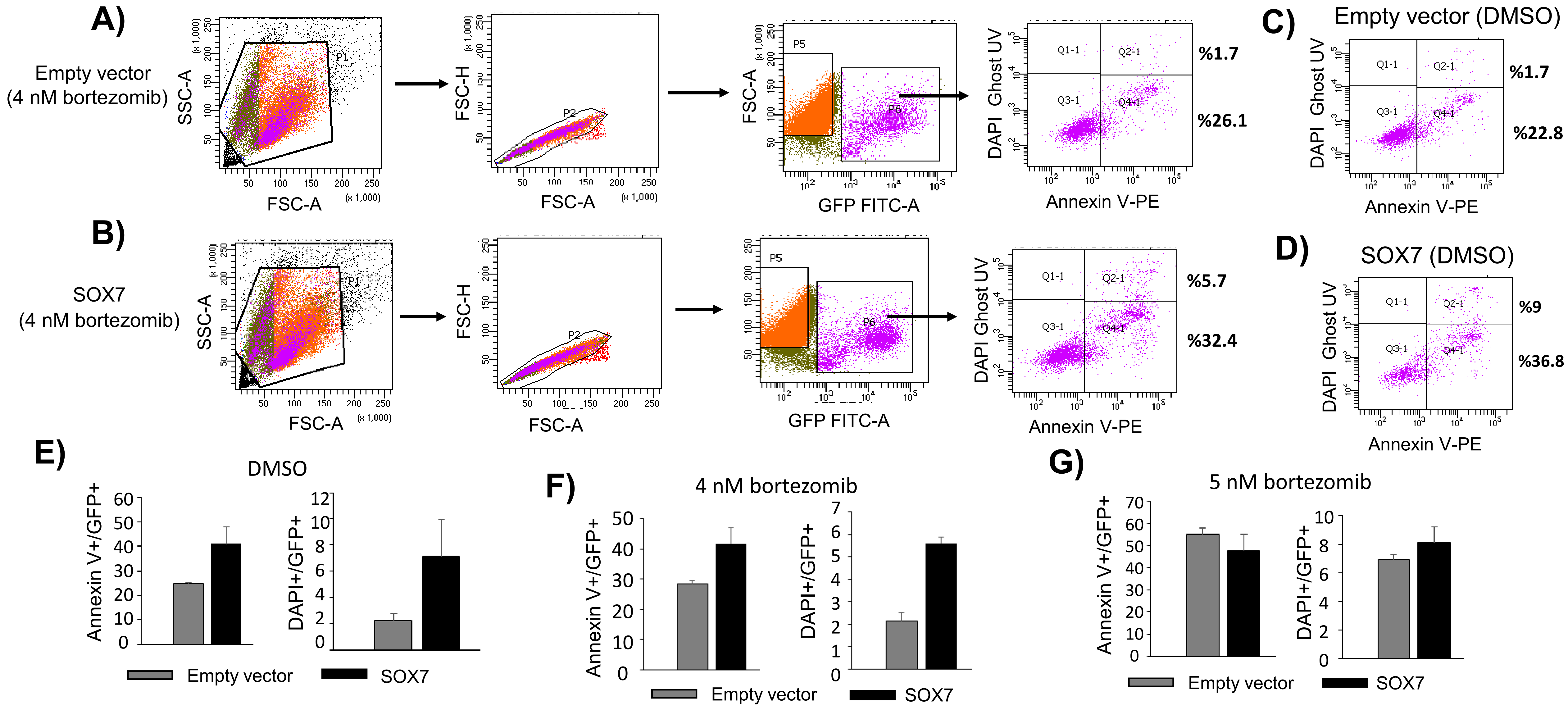

The effect of ectopic SOX7 on bortezomib sensitivity in MM.1S cells. Representative FACS gatings of empty vector or SOX7 transduced KMS-18 cells treated with 4 nM bortezomib (**A**,**B**) or only DMSO (**C**,**D**) for 48h. Bar graphs comparing Annexin V+ or DAPI+ cell percentages in EV or SOX7 transduced MM.1S cells in only DMSO (**E**), 4 nM bortezomib (**F**) or 5 nM bortezomib (**G**) treated MM.1S cells.

Figure S5

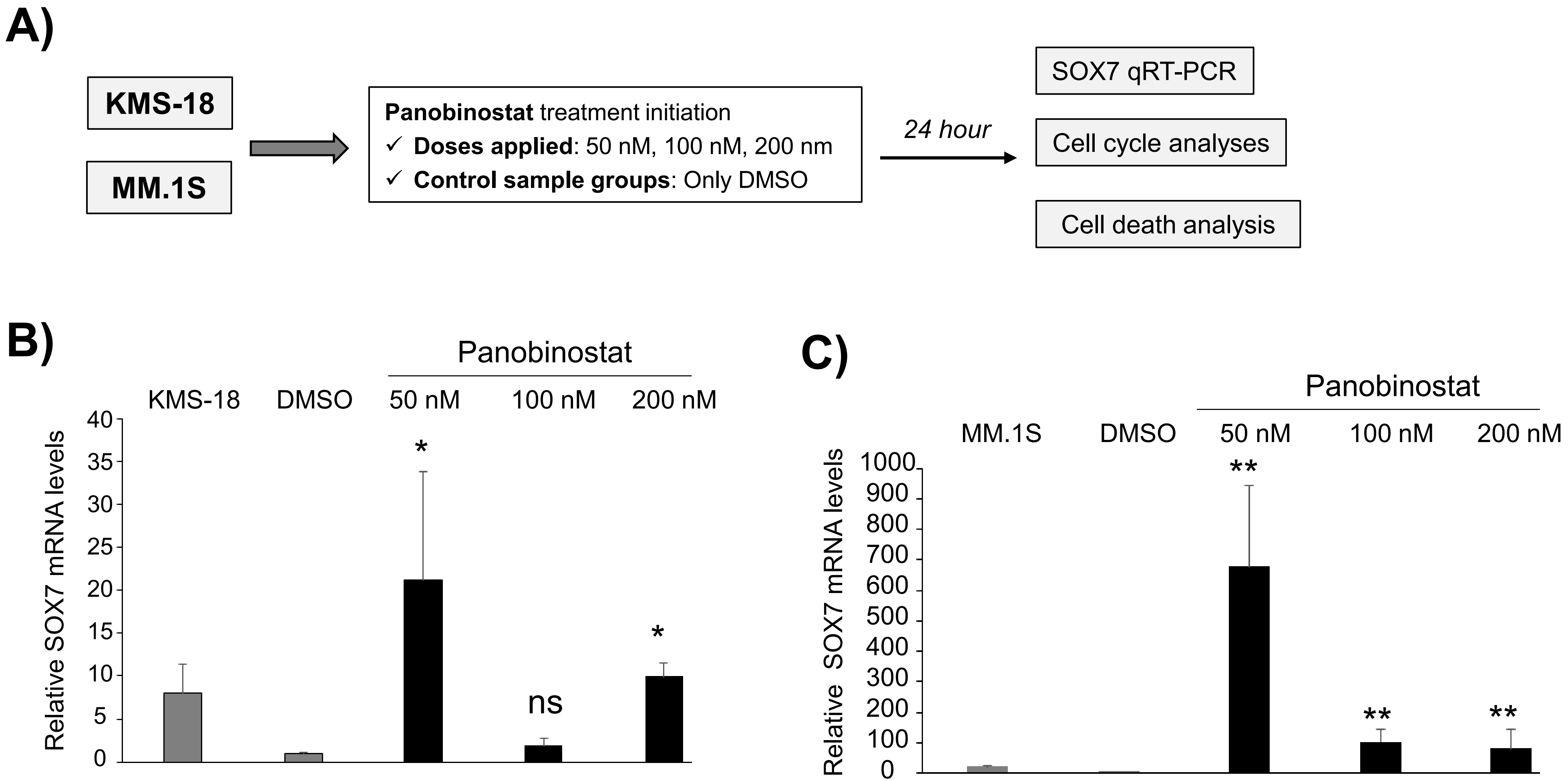

qRT-PCR results showing changes in SOX7 mRNA levels in KMS-18 and MM.1S treated with panobinostat. **A**) The workflow of panobinostat treatment experiments. SOX7 mRNA levels in KMS-18 (**B**) and MM.1S (**C**) treated with panobinostat 24h. SOX7 transcript levels were normalized to the levels in only DMSO treated control group. Average and standard deviation values represent four independent experiments each with replicate measurements. Gray colored bars show untreated samples; black colored bars show panobinostat treatment group. *: $p<0.05$, **: $p<0.001$ vs untreated or DMSO treated; ns: not significant.

Figure S6

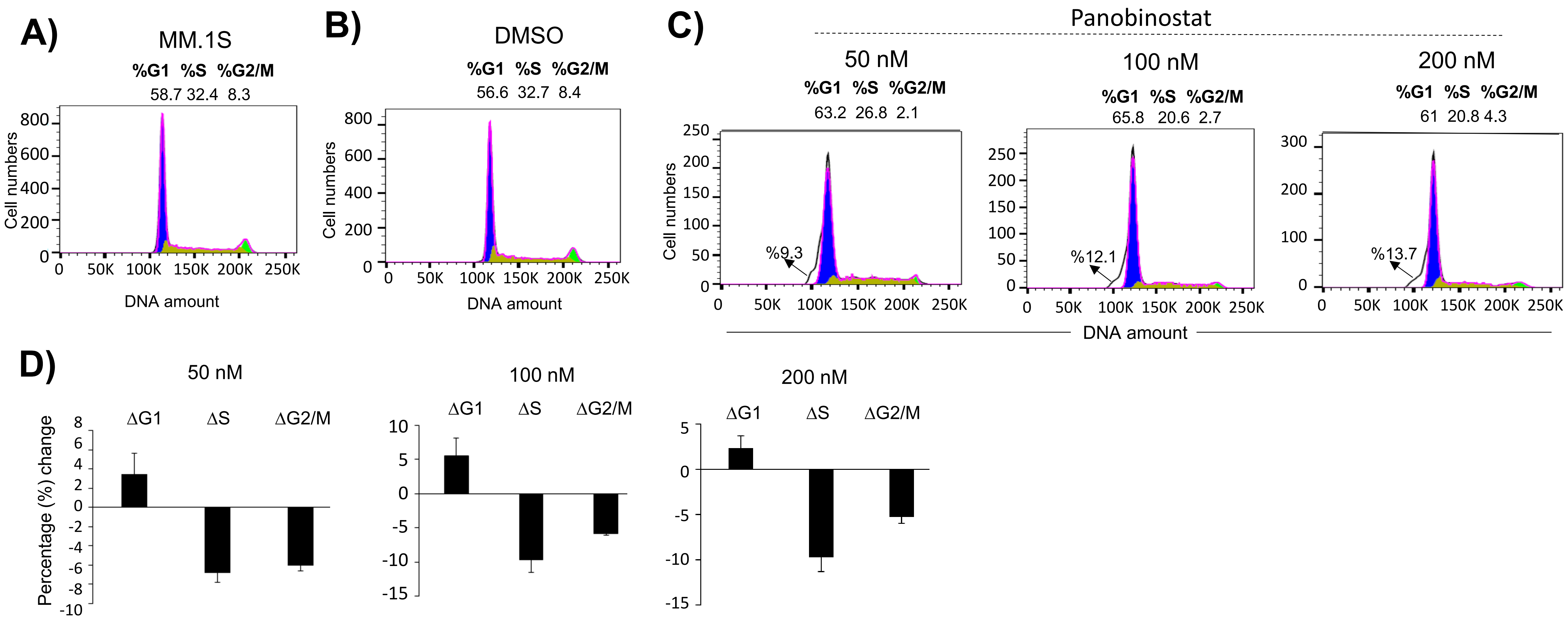

Panobinostat leads to G1 cell cycle arrest in MM.1S cells. The representative FACS plots showing percentages of cells in different cell cycle phases in untreated (**A**), only DMSO-treated (**B**), and panobinostat-treated (**C**) MM.1S cells. **D**) The graphs showing changes in cell cycle phases in MM.1S cells treated with 50 nM, 100 nM or 200 nM panobinostat compared with only DMSO treated cells. Average and standard deviation values of differences in cell cycle phases represent biological replicates of each sample group.

Figure S7

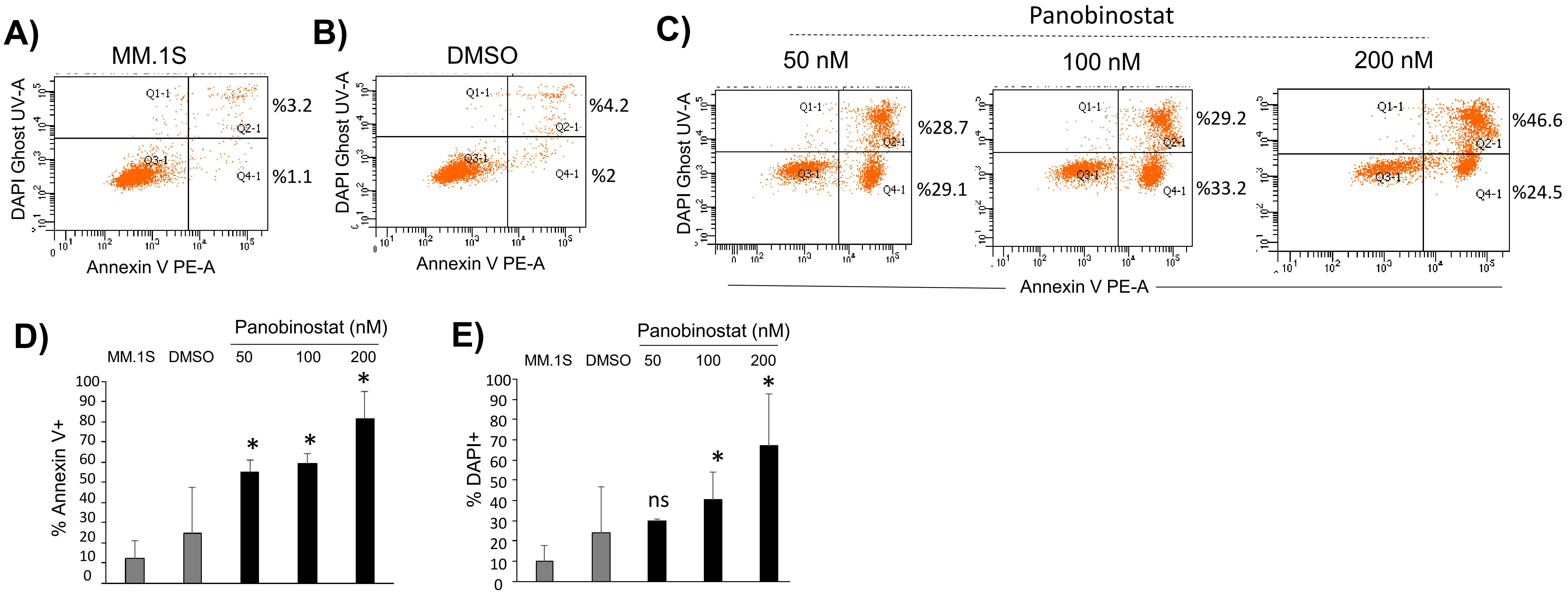

The effect of panobinostat on MM.1S cell survival. Representative FACS graphics showing Annexin V and DAPI staining in untreated (**A**), DMSO-treated (**B**), or panobinostat-treated (**C**) MM.1S cells. Bar graphs comparing annexin V (**D**) or DAPI (**E**) staining levels of control (gray) and panobinostat-treated (black) MM.1S cells. Average and standard deviation levels represent biological replicates of two independent experiments. *: $p<0.05$ vs untreated and DMSO; ns: Not significant.

Figure S8

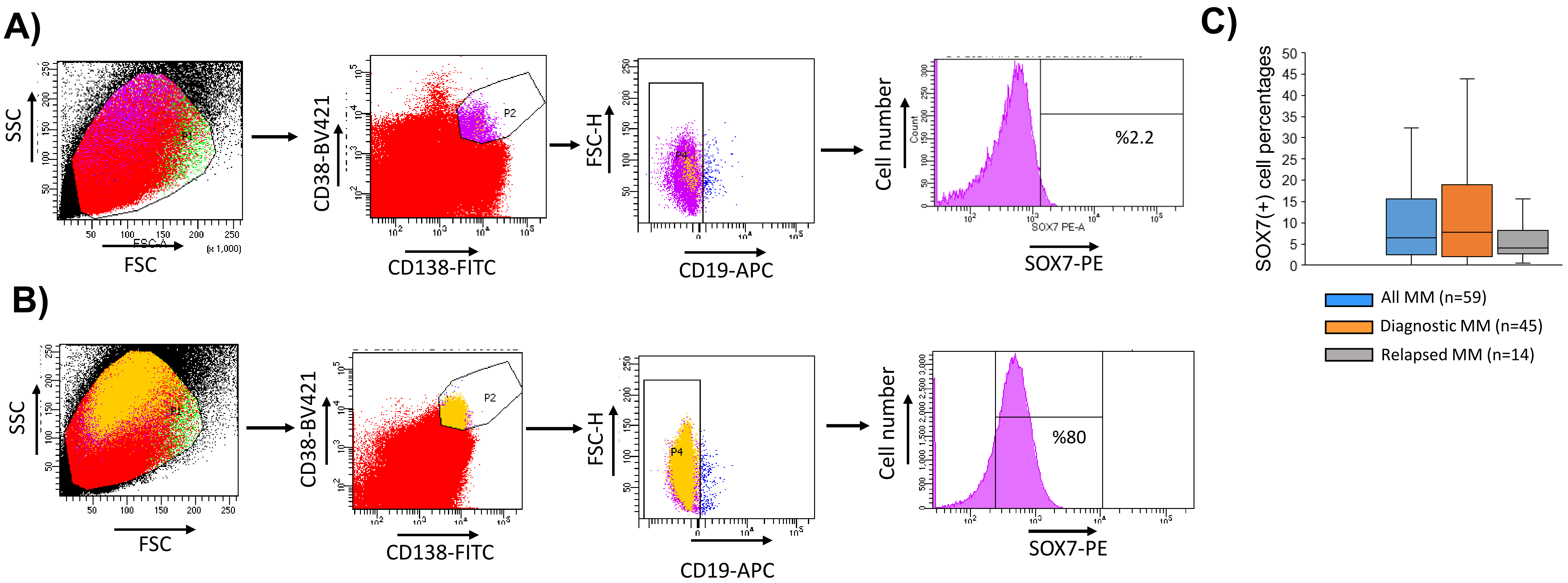

SOX7 protein levels in MM BM tumor cells in diagnostic and relapsed MM cases. The FACS gating steps followed during determination of SOX7 protein expression levels in representative MM BM tumor samples having low (**A**) and high (**B**) levels of SOX7 protein expression. **C)** Box-whisker graphic showing cell percentages expressing SOX7 protein in all, diagnostic, or relapsed MM cases.

Figure S9

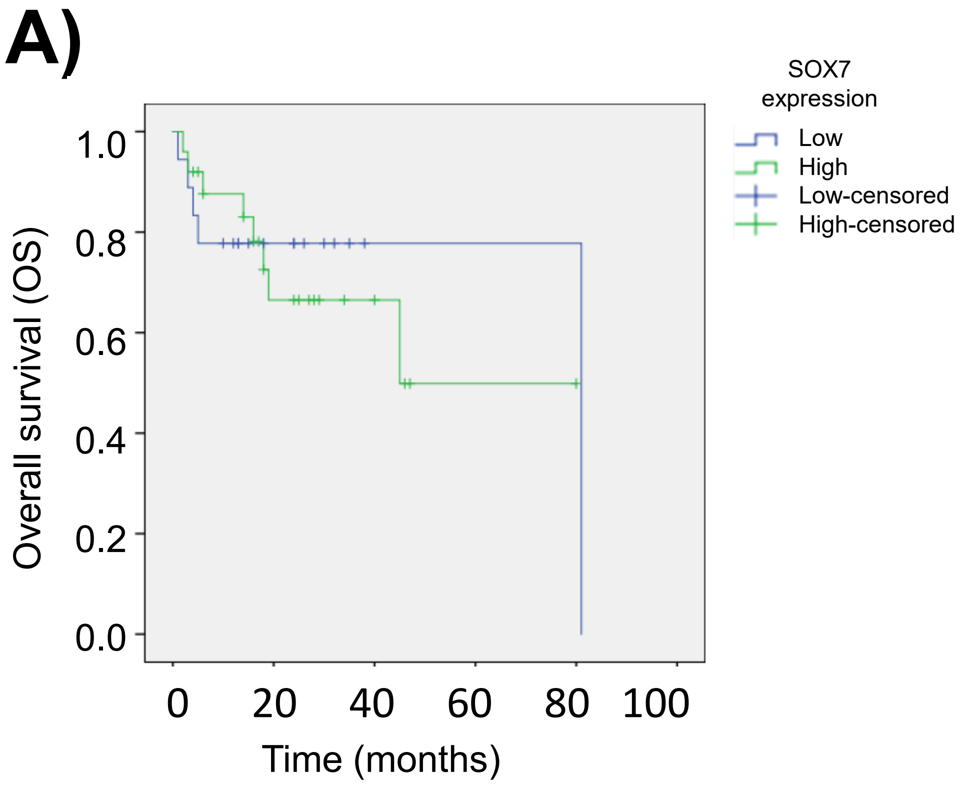

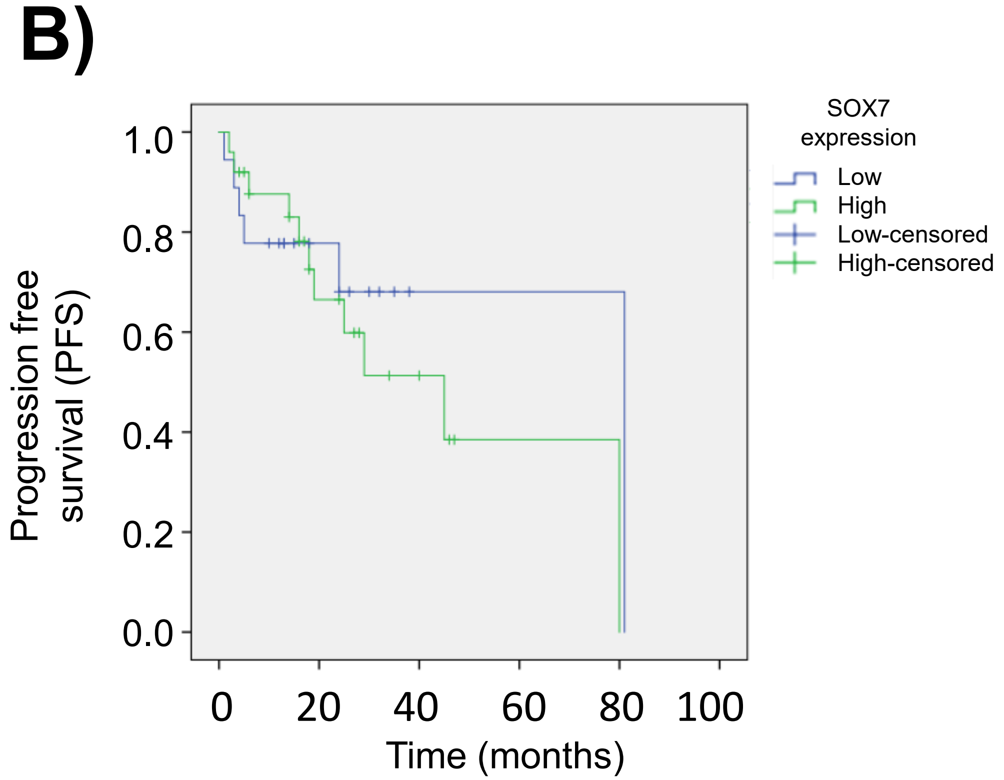

The relationship between SOX7 expression and MM patient survival. Kaplan Meier graphs showing SOX7 protein expression levels and overall survival (**A**) or progression free survival (**B**) of MM patients. **High**: MM patients with high SOX7 expression. **Low**: MM patients with low SOX7 expression.

Figure S10

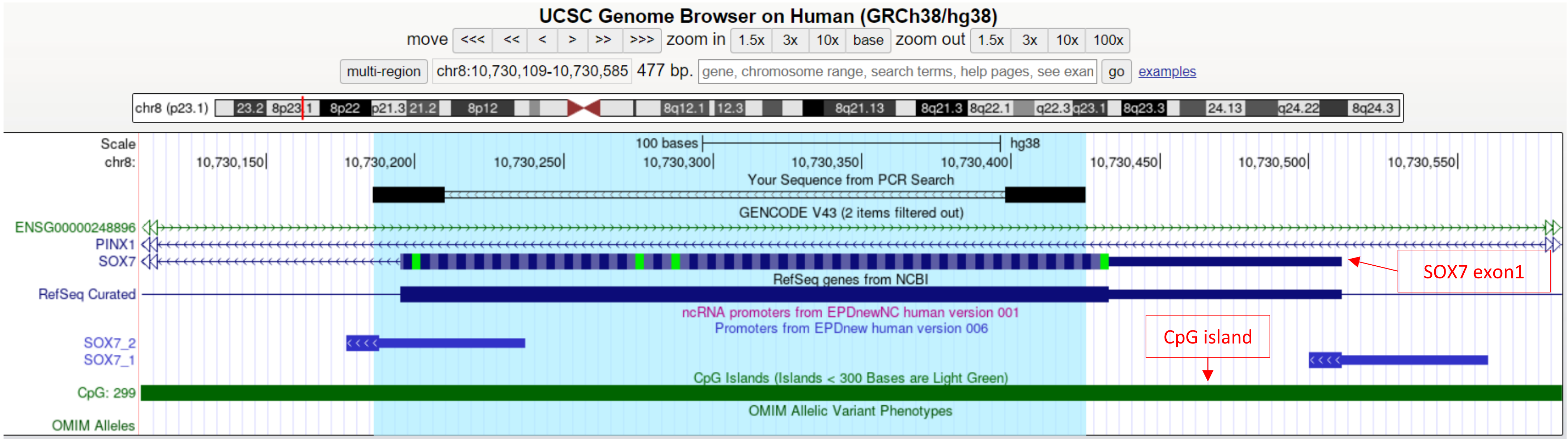

***SOX7* promoter region evaluated with bisulfite sequencing**. Blue shaded region represents the UCSC Genome Browser screen view for the part of the CpG island within 1st exon of SOX7, which was evaluated with bisulfite sequencing.

Figure S11

A)

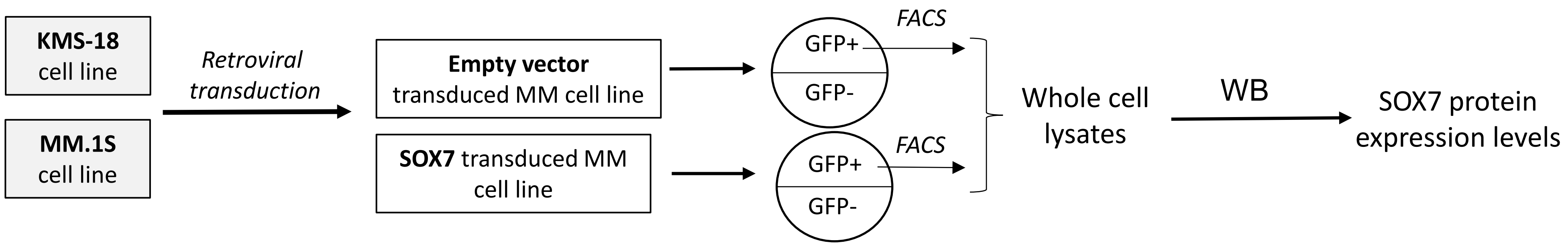

B)

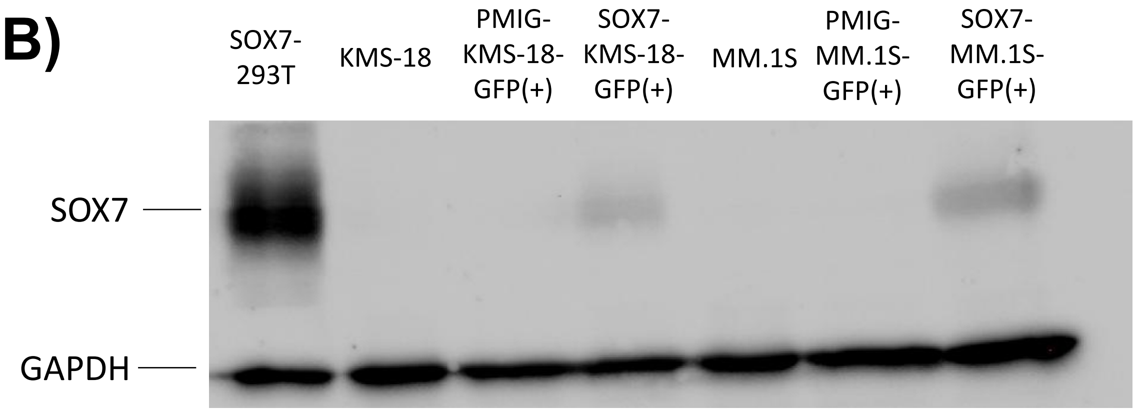

Ectopic expression of human SOX7 in MM cell lines. A) The workflow followed during determination of ectopic SOX7 protein expression in these MM cell lines. **B)** Western blot image showing ectopic SOX7 protein expression in KMS-18 and MM.1S cell lines whose GFP+ cells were FACS-sorted two days post-transduction. Ten µg whole cell lysate per each sample was run in SDS-PAGE gel. SOX7-transfected 293T cells (SOX7-293T) were used as the positive control. The two MM cell lines with/without empty vector transduction were used as negative controls.

Figure S12

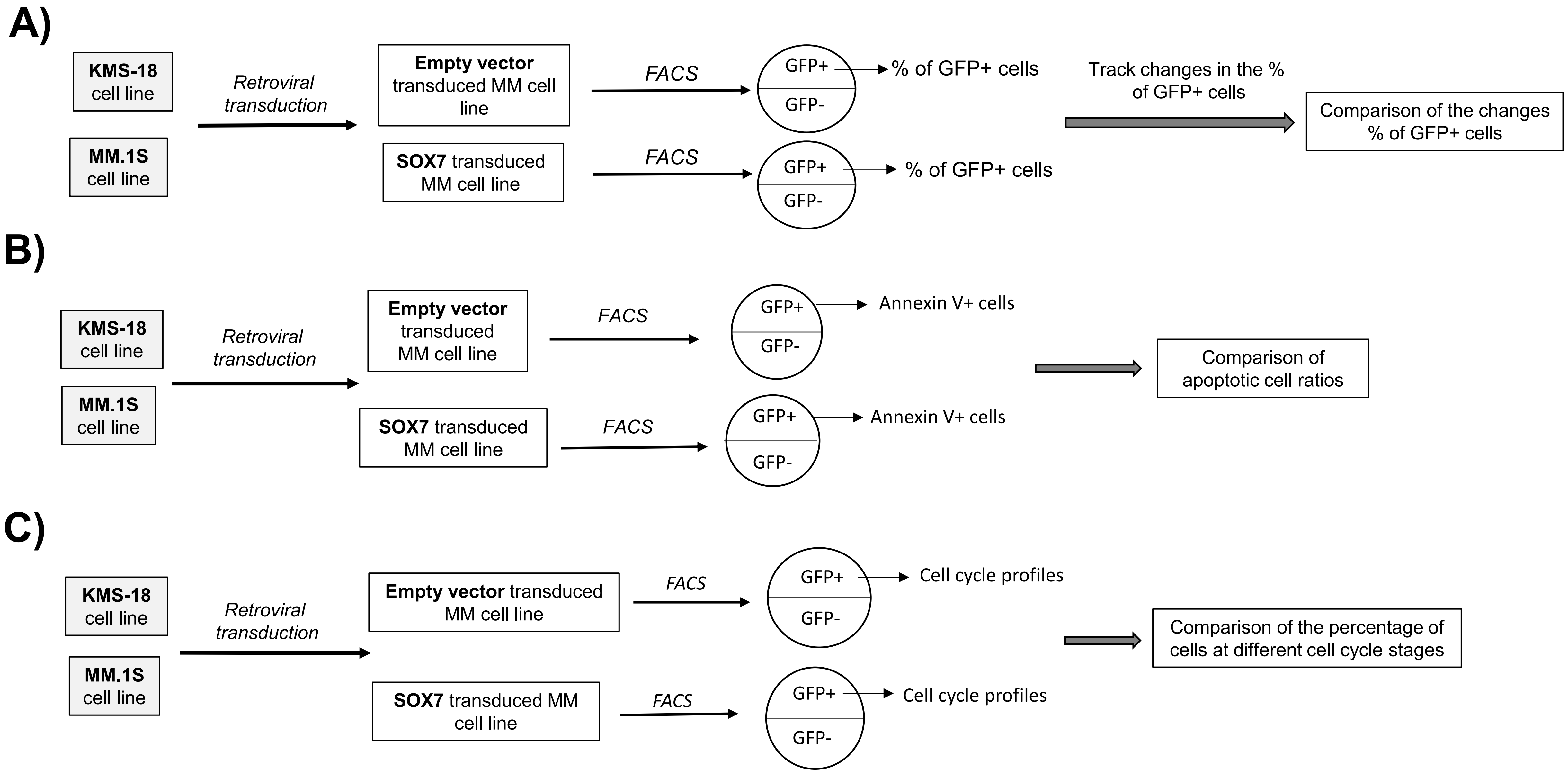

The experimental workflow of the GFP competition, cell cycle, and apoptosis assays.

Figure S13

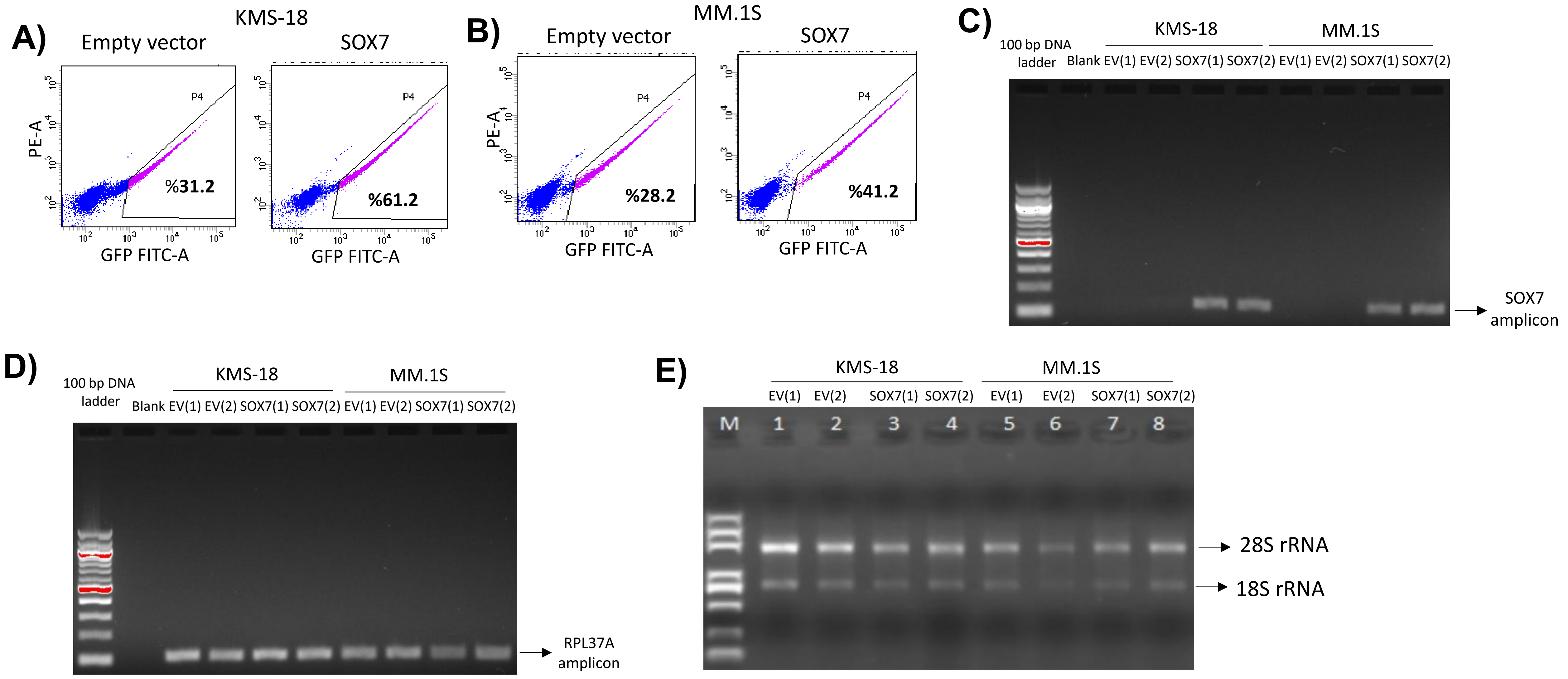

Quality control of MM cell lines with ectopic SOX7 expression before WTS. The representative FACS graphs of KMS-18 (**A**) and MM.1S (**B**) cell line two days post-transduction with PMIG or SOX7-PMIG. The TAE gel images of SOX7 (**C**) or RPL37A (**D**) amplicons of GFP+ cells FACS-sorted two days post-transduction with empty vector or SOX7. **E**) The image of total RNA samples run through TAE agarose gel. **EV**: Empty vector; **SOX7**: SOX7-PMIG; **M**: DNA ladder.
