# Supplementary material for: Comprehensive Analyses of SOX7 Provide Novel Insights on Its Tumor Suppressor Role and Its Target Genes with Therapeutic Implications in Multiple Myeloma
Source: Int J Mol Sci. 2026 Aug 26;27(17):7648. doi: 10.3390/ijms27177648 (PMC13566877; doi:10.3390/ijms27177648)
Supplement: Supplementary file 1 [file ijms-27-07648-s001.zip › Table S3. ChIP-Seq NGS quality control stats (IJMS).pdf]

Table S3. ChIP-Seq NGS quality control statistics

Sample	Raw reads	Raw data	Effective (%)	Error (%)	Q20 (%)	Q30 (%)	GC (%)
SOX7-MM.1S (1)	31787502	4.8	38.77	0.02	95.39	89.81	54.48
PMIG-MM.1S (1)	48714078	7.3	87.50	0.01	98.36	95.88	59.52
SOX7-MM.1S (2)	53314422	8.0	17.76	0.02	93.46	84.06	58.90
PMIG-MM.1S (2)	48682654	7.3	11.43	0.03	92.87	81.74	59.30
SOX7-KMS18 (1)	174679020	26.2	20.30	0.02	93.29	83.41	60.14
PMIG-KMS18 (1)	47794844	7.2	27.00	0.02	94.71	86.76	56.90
SOX7-KMS18 (2)	51778906	7.8	24.48	0.02	93.88	84.86	50.34
PMIG-KMS18 (2)	63695034	9.6	27.70	0.02	94.02	84.97	59.48
