# Supplementary material for: Comprehensive Analyses of SOX7 Provide Novel Insights on Its Tumor Suppressor Role and Its Target Genes with Therapeutic Implications in Multiple Myeloma
Source: Int J Mol Sci. 2026 Aug 26;27(17):7648. doi: 10.3390/ijms27177648 (PMC13566877; doi:10.3390/ijms27177648)
Supplement: Supplementary file 1 [file ijms-27-07648-s001.zip › Table S4. WTS NGS quality control stats (IJMS).pdf]

Table S4. Quality control results of whole tanscriptome sequencing

Sample	Raw reads	Raw data	Effective(%)	Error(%)	Q20(%)	Q30(%)	GC(%)
KMS18_SOX7_1	94838814	15.3	99.09	0.03	97.73	93.71	43.99
	7401994		99.16	0.03	97.84	93.91	43.91
KMS18_PMIG_1	100414534	15.1	98.67	0.03	97.53	93.21	44.66
KMS18_SOX7_2	122503062	18.4	98.87	0.03	97.34	92.65	42.55
KMS18_PMIG_2	112043484	16.8	98.89	0.03	97.75	93.82	44.31
MM1S_SOX7_1	126580106	19.0	98.87	0.03	97.52	93.21	43.92
MM1S_PMIG_1	96955496	15.5	98.93	0.03	97.75	93.72	44.53
	6409006		99.00	0.03	97.65	93.42	44.48
MM1S_SOX7_2	91230520	15.9	98.43	0.03	97.76	93.73	44.04
	14794078		98.49	0.03	97.69	93.54	44.08
MM1S_PMIG_2	123273072	18.5	98.97	0.03	97.65	93.49	44.80

Sample: Sample name
Raw reads: Total amount of reads of raw data, each four lines taken as one unit. For paired-end sequencing, it equals the amount of read1 and read2, otherwise it equals the amount of read1 for single-end sequencing.
Raw data: (Raw reads) * (sequence length), calculating in G. For paired-end sequencing like PE150, sequencing length equals 150, otherwise it equals 50 for sequencing like SE50.
Effective: (Clean reads/Raw reads)*100%
Error: Base error rate
Q20, Q30: (Base count of Phred value > 20 or 30) / (Total base count)
GC: (G & C base count) / (Total base count)
